# GLUT3 inhibitor discovery through in silico ligand screening and in vivo validation in eukaryotic expression systems

**DOI:** 10.1038/s41598-022-05383-9

**Published:** 2022-01-26

**Authors:** Cristina V. Iancu, Giovanni Bocci, Mohd Ishtikhar, Moumita Khamrai, Mislav Oreb, Tudor I. Oprea, Jun-yong Choe

**Affiliations:** 1 East Carolina Diabetes and Obesity Institute, East Carolina University, Greenville, NC 27834 USA; 2 Translational Informatics Division, Department of Internal Medicine, The University of New Mexico School of Medicine, Albuquerque, NM 87131 USA; 3 Institute of Molecular Biosciences, Faculty of Biological Sciences, Goethe University Frankfurt, Frankfurt am Main, Germany; 4 UNM Comprehensive Cancer Center, The University of New Mexico, Albuquerque, NM 87131 USA; 5 Department of Rheumatology and Inflammation Research, Institute of Medicine, Sahlgrenska Academy, University of Gothenburg, Gothenburg, Sweden; 6 Novo Nordisk Foundation Center for Protein Research, Faculty of Health and Medical Sciences, University of Copenhagen, Copenhagen, Denmark; 7 Department of Chemistry, East Carolina University, Greenville, NC 27834 USA; 8 Department of Biochemistry and Molecular Biology, Rosalind Franklin University of Medicine and Science, North Chicago, IL 60064 USA

**Keywords:** Membrane proteins, Virtual screening, Analytical biochemistry, Dietary carbohydrates

## Abstract

The passive transport of glucose and related hexoses in human cells is facilitated by members of the glucose transporter family (GLUT, SLC2 gene family). GLUT3 is a high-affinity glucose transporter primarily responsible for glucose entry in neurons. Changes in its expression have been implicated in neurodegenerative diseases and cancer. GLUT3 inhibitors can provide new ways to probe the pathophysiological role of GLUT3 and tackle GLUT3-dependent cancers. Through in silico screening of an ~ 8 million compounds library against the inward- and outward-facing models of GLUT3, we selected ~ 200 ligand candidates. These were tested for in vivo inhibition of GLUT3 expressed in hexose transporter-deficient yeast cells, resulting in six new GLUT3 inhibitors. Examining their specificity for GLUT1-5 revealed that the most potent GLUT3 inhibitor (G3iA, IC_50_ ~ 7 µM) was most selective for GLUT3, inhibiting less strongly only GLUT2 (IC_50_ ~ 29 µM). None of the GLUT3 inhibitors affected GLUT5, three inhibited GLUT1 with equal or twofold lower potency, and four showed comparable or two- to fivefold better inhibition of GLUT4. G3iD was a pan-Class 1 GLUT inhibitor with the highest preference for GLUT4 (IC_50_ ~ 3.9 µM). Given the prevalence of GLUT1 and GLUT3 overexpression in many cancers and multiple myeloma’s reliance on GLUT4, these GLUT3 inhibitors may discriminately hinder glucose entry into various cancer cells, promising novel therapeutic avenues in oncology.

## Introduction

Glucose is essential for the cells of most organisms as a source of energy and precursors for cellular building blocks, including lipids and amino acids. In humans, glucose diffusion through the cellular membrane is facilitated by members of the glucose transporter family (GLUT, SLC2 gene family)^1^. The 14 GLUT isoforms are grouped according to protein sequence similarity into three classes: Class 1 (GLUT1-4, 14), Class 2 (GLUT5, 7, 9, and 11), and Class 3 (GLUT6, 8, 10, 12, and 13 or HMIT)^1,2^. Most GLUTs transport glucose and/or fructose, though with different affinity, while GLUT9 (SLC2A9) transports uric acid and fructose, and HMIT (SLC2A13) is a proton myo-inositol symporter. Differences in GLUT tissue expression accord with local physiological needs and reflect variations in GLUT substrate affinity and specificity^2^. Alterations in the activity or expression of GLUTs have been implicated in Mendelian disorders (e.g., GLUT1 deficiency and Fanconi-Bickel syndrome)^3,4^, cancer^5^, diabetes^6^, obesity^7^, renal disease^8^, neurodegenerative diseases^9^, and the metabolic syndrome^10^. Therefore, GLUT-specific inhibitors provide valuable investigative tools for the pathophysiological roles of these essential transporters, especially in tissues expressing GLUTs with overlapping activities, and potential therapeutic approaches to combat GLUT-related diseases.

All Class 1 GLUTs transport glucose, though GLUT2 can also transport fructose. GLUT3 is the highest affinity glucose transporter (2-deoxy-D-glucose K_M_ ~ 1.4 mM) and is expressed primarily in neurons^1,11,12^. The level of GLUT3 correlates with regional brain glucose utilization^13^. A characteristic feature of Alzheimer’s disease (AD) is the progressive reduction of cerebral metabolic rate for glucose^14^. In the AD brain, GLUT3 level is decreased^15^ and correlates with the extent of tau hyperphosphorylation and the density of neurofibrillary tangles^16^. However, the mechanism of GLUT3 reduction in the AD brain remains unclear.

Given the cancer cells’ high dependence on glucose metabolism and inherent metabolic reprogramming, most cancers show changes in the pattern and level of GLUT expression, with some GLUTs being expressed in tissues where they normally lack and native GLUTs being significantly upregulated^5^. In F^18^-deoxy-glucose positron emission tomography (FDG-PET) cancer diagnosis, tumors are visualized as sites that concentrate the glucose analog FDG through the activity of GLUTs^17^. Hypoxia, a major characteristic of solid tumors, is associated with resistance to chemo- and radiotherapies^18,19^, and drives the overexpression of GLUT1 and GLUT3^20,21^. GLUT inhibition can reverse the tumor resistance to therapies, enhancing the chemosensitivity to anticancer drugs^22,23^. GLUT1 overexpression occurs in many cancers, including lung^24^, prostate^25^, breast^26^, colorectal^27^, gastric^28^, kidney^29^, and other cancers^30^. It was proposed as a prognostic marker as it often indicates tumor invasiveness and poor outcomes^31^. Thus, GLUT1 is an actively pursued cancer target.

GLUT3 is also overexpressed in several cancers, and its upregulation correlates with poor survival and tumor aggressiveness in brain^32,33^, lung^34,35^, laryngeal^36^, breast^37^, gastric^38^, liver^39^, and colorectal^40^ cancers. In acute myeloid leukemia cells, silencing GLUT3 results in strong cell growth inhibition and apoptosis^41^. Moreover, the cells became resensitized to vincristine, a chemotherapy drug, indicating synergy between GLUT3 inhibition and vincristine. Epithelial-mesenchymal transition can endow neoplastic properties to cells, being critical to cancer invasiveness and dissemination^42^. GLUT3 is overexpressed in mesenchymal but not epithelial, liver and lung cells, and its inhibition can provide a targeted therapy of poorly differentiated tumors^35^. Hence, GLUT3 shows promise as a therapeutic target in oncology.

GLUT4 overexpression has been reported in colon, breast, thyroid, pancreatic, and gastric carcinomas^43–47^. Multiple myeloma relies on GLUT4^48^, prompting searches for GLUT4-selective inhibitors as anticancer drugs^49^. GLUT4 inhibitors have cytotoxic and chemosensitizing effects on multiple myeloma cell lines and patient samples, validating the role of GLUT4 as an oncologic target. In summary, ligands with defined inhibitory activity for GLUT1, GLUT3, and GLUT4 can debilitate various cancer cells by depriving them of energy or sensitizing them to anticancer therapies.

Human GLUTs belong to the major facilitator superfamily (MFS), one of the largest and most ubiquitous families of membrane proteins^50,51^. Most MFS proteins share a topology of 12 transmembrane helices, organized as two pseudosymmetric halves that nestle the substrate cavity^52^. Proposed transport mechanisms for MFS proteins include the alternate access mechanism, in which the substrate cavity is alternately exposed to the outside (outward-facing or exofacial conformation) or inside (inward-facing or endofacial conformation) of the cell^53^, and the fixed-site transporter with co-existing exo and endofacial conformations^54,55^, as advanced for GLUT1^55^. Crystal structures of several human GLUTs (GLUT1^56^, GLUT3^57^, and GLUT5^58^) and their homologs (XylE^59,60^, GlcP_Se_^61^) have captured the outward- and inward-facing conformations, with the substrate binding site accessible (open state) or shielded (occluded state). These structures facilitate homology structure modeling of GLUT isoforms for computational ligand screening, enabling the discovery of GLUT-discerning ligands^49,62,63^. Depending on which transporter conformation is used for target-based virtual screening (TBVS)^64^, such an approach identifies ligands that bind to the extracellular or cytosolic part of the transporter cavity. TBVS with the inward-facing conformation of GLUT5 (modeled based on GlcP_Se_^61^) and GLUT2 (modeled based on GLUT1^56^) identified the first potent and specific inhibitors for these transporters^62,63^. Establishing the ligand selectivity among closely related GLUT isoforms requires systems that assay a single GLUT. Such GLUT-specific assay systems have been established in hexose transporter-deficient yeast strains engineered to express a single GLUT and are available for GLUT1-5^65–67^. Their application to GLUT ligand discovery and selectivity assessment has been recently demonstrated by Schmidl et al.^63^, who have reported eleven novel GLUT2 inhibitors, among which nine are GLUT2-specific.

GLUT-discerning ligands provide valuable investigative tools for the pathophysiological roles of these essential transporters and potential therapeutic approaches to combat GLUT-related diseases, including cancer and metabolic disorders. Most known GLUT3 inhibitors (e.g., cytochalasin B^68^, phloretin^69^, quercetin^69^, etc.) also affect other Class 1 GLUTs. Here, we combined TBVS using the inward- and outward-facing GLUT3 conformations with the yeast assay platform studies for individual GLUTs to identify novel GLUT3 inhibitors and assess their selectivity for the other Class 1 GLUTs and GLUT5.

## Results

### Target-based virtual screening (TBVS)

Inhibitor discovery by TBVS depends on the library of small compounds used, the structural model (including transporter conformation), and the docking methods. Crystal structures of GLUTs and their homologs show two major transporter conformations, the inward- and outward-facing conformations^56–61,70^. Mutations or ligands that lock the transporter in one conformation lead to loss of transport activity^70,71^. For example, cytochalasin B, a potent inhibitor of GLUT1 and other GLUTs, inhibits the transport by binding to the inward-facing conformation of the transporter^70^. Previously, we applied TBVS to inhibitor discovery for GLUT2 and GLUT5, using the inward-facing conformation models of these transporters and ligand binding pockets that spanned the substrate binding site (GLUT5) or the substrate cavity entrance, excluding the active site (GLUT2)^62,63^.

**Figure 1:**
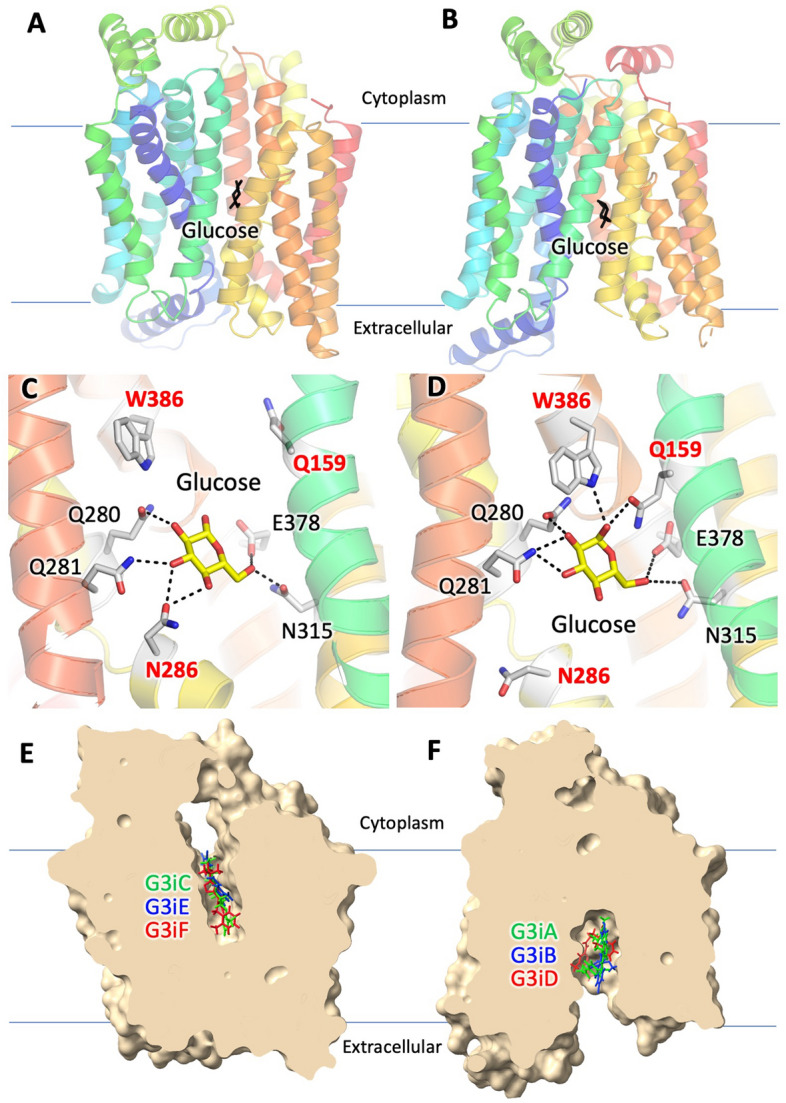
Models of the inward- and outward-facing conformations of GLUT3 showing the sites of selected docked ligands from the target-based virtual screening (TBVS). Overview of the GLUT3 models for the inward-facing **(A)** and outward-facing **(B)** conformations showing the glucose binding site (glucose as stick model, colored black). The rainbow coloring of the protein ribbon diagram starts with blue at the N-terminus and ends with red at the C-termius. Close-up of the glucose binding site in the inward-facing **(C)** and outward-facing **(D)** conformations of GLUT3. Glucose modeling was based on the glucosyl moiety of nonyl beta-D-glucopyranoside from PDB ID 4PYP **(C)** and alpha-D-glucopyranose-(1–4)-alpha-D-glucopyranose from PDB ID 4ZWC **(D)**. Glucose (yellow) and active site residues (grey) are shown as stick models. Dashed black lines represent hydrogen bond interactions. Side chains of Q159, N286 and W386 (red labels) adopt different conformations in the two conformations. Central slices through the GLUT3 isosurface showing the targeted ligand pockets in the inward-facing **(E)** and outward-facing **(F)** conformations. Validated ligand candidates from TBVS are shown as stick models, colored from the highest to the lowest potency as green, blue, and red (see also Table 1). **(A,C,E)** Homology model for the GLUT3 inward-facing conformation was generated in MOE, using as a template the crystal structure of GLUT1 (PDB ID 4PYP). **(B,D,F)** Structural model for the outward-facing conformation was the GLUT3 crystal structure PDB ID 5C65. The figures were generated with Pymol (https://www.pymol.org) **(A–E)** and ChimeraX (https://www.rbvi.ucsf.edu/chimerax) **(E–F)**.

Here, we screened the ChemNavigator library containing ~ 8 million commercially available chemicals against both major conformations of GLUT3, targeting the substrate binding site. Some side-chain conformations of residues in the glucose binding site (e.g., Q159, N286, and W386) differ in the two transporter conformations (Fig. 1C,D). These differences combined with distinct protein regions in the active site proximity for the two conformations produce different ligand binding pockets (Fig. 1E,F).

So far, crystal structures for GLUT3 captured only the outward-facing conformation^57^, while those for GLUT1 are in the inward-facing conformation^56,70^. The outward-facing conformation model was that of the GLUT3 crystal structure PDB ID 5C65. For the inward-facing conformation of GLUT3, we generated a homology model based on the inward-facing conformation of GLUT1 (PDB ID 4PYP), which shares 66% sequence identity^57^ with GLUT3. To implement the structure-based virtual screening (SBVS) procedure, we used the software FLAP (Fingerprints for Ligands and Proteins)^72^. First, we extracted the substrate binding site from the outward- and inward-facing conformations. Second, we used both cavities to screen the compound library. Amongst various similarity scores computed by FLAP, the Glob-Prod (GP) score measures the overall GRID fields similarity between the binding site and the screened compound. This similarity score estimates the likelihood of interaction between a cavity and a ligand. Thus, we used the GP score to rank the compounds after separately screening them with both the inward- and outward-facing GLUT3 conformations. Among the top-ranked 200 ligand candidates, 100 from the screening of each GLUT3 conformation model, 193 were purchased to be tested (Supplementary Table S1). Figure 1 shows an overview of the docking sites for some ligand candidates to the outward- and inward-facing GLUT3 models.

### Screening of GLUT3 ligand candidates for inhibition of GLUT3 transport activity

To experimentally validate the ligand candidates determined by the virtual ligand screening, we tested their effect on GLUT3 transport activity. For this, we used the recently established assay system^67^ in which GLUT3 is expressed in a yeast strain devoid of endogenous hexose transporters (*hxt*^*0*^) so that glucose uptake into cells relies solely on the activity of GLUT3. Similar assay systems are available for GLUT1^65^ , GLUT2^67^, GLUT4^65^, and GLUT5^66^, enabling identification and selectivity assessment of their inhibitors^63^.

We determined GLUT3 transport activity by measuring the accumulation of radioactive glucose inside whole *hxt*^*0*^ yeast cells (see “Materials and methods” for details). To select an appropriate substrate concentration for transport inhibition screening, we assessed the K_M_ of glucose for GLUT3 in *hxt*^*0*^ cells. Its value of 3.11 ± 0.42 mM (Supplementary Fig. S1) is higher than that for 2-deoxy-glucose reported for GLUT3 expressed in *Xenopus laevis* oocytes (K_M, 2-deoxy-glucose_ ~ 1.4 mM^12^), the discrepancy possibly stemming from the different structure of the substrates or the changed lipid environment. Indeed, functional expression of human GLUTs in *hxt*^*0*^ cells often requires mutations in genes related to yeast lipid composition^65,73^ as well as single-site mutations that may favor the outward-facing conformation of the transporters^65–67^. As previously proposed^69^, this suggests that the transporter conformational dynamics depends on the lipid environment and, in the yeast membrane, GLUT’s function is ‘nativized’ by facilitating the outward-facing conformation. For GLUT3 as well, compared to the wild-type, S66Y mutation had a better glucose transport activity (Supplementary Fig. S1), as indicated also by the more vigorous cell growth in glucose-based media^67^. Moreover, we found that GLUT3_S66Y_ transports glucose with K_M_ = 1.42 ± 0.17 mM, which is ~ twofold better than that of the wild-type (Supplementary Fig. S1). For inhibitor screening, we used the wild-type GLUT3 in *hxt*^*0*^ cells and measured the transport activity at 3 mM glucose concentration, to approximate its K_M_ value in the yeast-based system.

**Figure 2:**
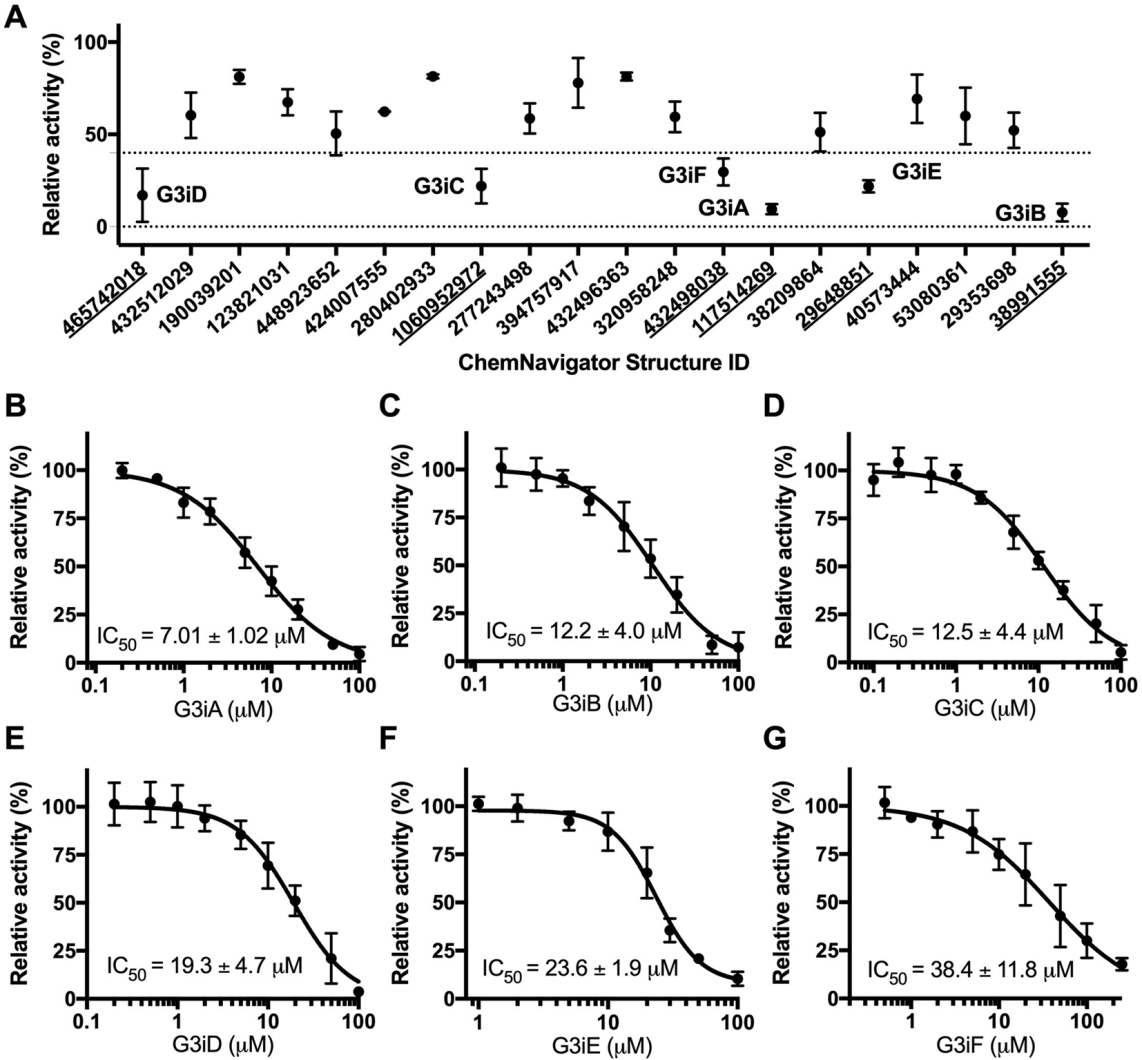
Effect of compounds identified from TBVS on GLUT3 transport activity. **(A)** GLUT3 relative transport activity in the presence of 50 µM compound concentration, at 3 mM glucose concentration. Glucose transport activity of GLUT3 expressed in *hxt*^*0*^ yeast cells EBY.S7 was measured as C^14^-glucose uptake in whole cells (see “Materials and methods” for details). The compounds are identified by the ChemNavigator Structure ID. Six compounds, G3iA–G3iF (ChemNavigator Structure ID number underlined), inhibited GLUT3 relative activity by more than 60%. Initial screening at 100 µM concentration for all 193 tested compounds (listed in Supplementary Table S1) is shown in Supplementary Fig. S2. **(B–G)** Dose response curves for G3iA-G3iF inhibition of GLUT3 relative transport activity. Standard deviations for experimental points come from at least three independent measurements. Graphs, data analysis, and IC_50_ values were generated with GraphPad (https://www.graphpad.com).

The initial activity screening of the GLUT3 ligand candidates was performed at 100 µM compound concentration (Supplementary Fig. S2). Among 193 ligand candidates, we found that 20 decreased the GLUT3 transport activity by more than 60%, and we re-screened them at 50 µM concentration (Fig. 2A). This time, six compounds inhibited the GLUT3 transport activity by more than 60% and were further examined to determine their IC_50_ for transport inhibition in the wild-type and S66Y GLUT3 *hxt*^*0*^ yeast systems (Fig. 2B–G, Supplementary Fig. S3). Within the margin of error, the inhibitor IC_50_ values determined with the two assay systems were similar, though GLUT3_S66Y_ seemed consistently better inhibited than the wild type (Supplementary Figure S3, Supplementary Table S2). The identified GLUT3 inhibitors had IC_50_ values between ~ 7 and 38 µM for GLUT3 (Fig. 2, Table 2), and between ~ 6 and 25 µM in GLUT3_S66Y_ (Supplementary Fig. S3, Supplementary Table S2). We named this compound series G3i (“GLUT3 inhibitor”) A–F in the order of decreasing inhibition potency (Table 1, Fig. 2, Supplementary Table S1). The G3iA, G3iB, and G3iD inhibitors were identified from TBVS using the outward-facing conformation, while G3iC, G3iE, and G3iF were identified from TBVS against the inward-facing conformation (Fig. 1). Since the inhibitors targeted the glucose binding site in the TBVS, we expected them to be competitive with glucose. We checked the inhibition mode for G3iA and G3iC, the most potent GLUT3 inhibitors for each GLUT3 conformation (Fig. 1, Table 1). Both inhibitors were competitive with glucose (K_i, G3iA_ = 3.49 ± 0.47 µM, and K_i,G3iC_ = 5.83 ± 0.30 µM, Supplementary Fig. S4A,B). Accordingly, the TBVS-predicted docking sites for G3iA and G3iC partially or fully occupied the glucose binding site (Supplementary Fig. S5A,C). Also, the predicted docking sites for G3iB, G3iD, and G3iF overlapped with the glucose binding site, whereas that of G3iE was in sufficiently close proximity to the active site to engage well-known glucose interacting residues such as Q159, Q280, and W386^1,57^ (Supplementary Fig. S5, Fig. 4E). Indeed, G3iE, also exhibited competition with glucose (K_i,G3iE_ = 12.3 ± 0.9 µM, Supplementary Fig. S4C).

**Table 1:**
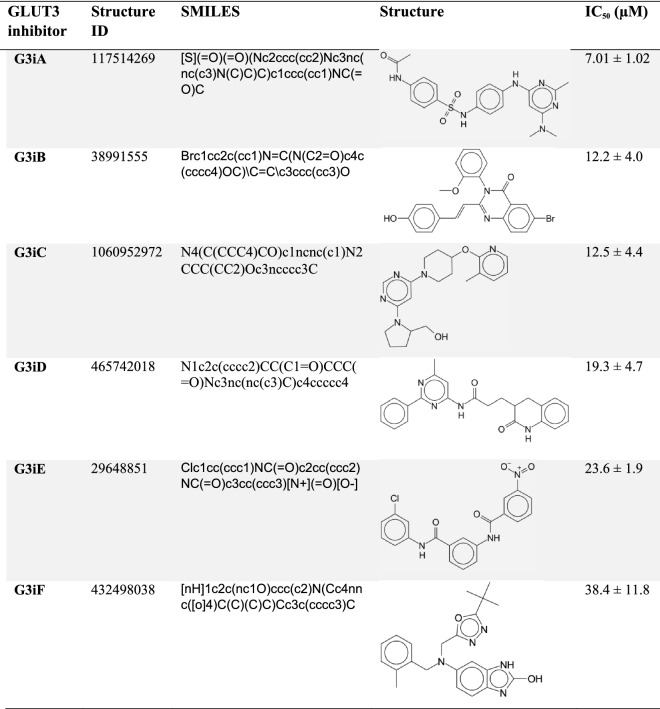
Structures of GLUT3 inhibitors.

For each GLUT3 inhibitor, the ChemNavigator structural ID, SMILES code, and IC_50_ values for GLUT3 inhibition are indicated. Corresponding dose–response curves are in Fig. 2B–G. G3iA, G3iB, and G3iD are inhibitors resulted from TBVS of the outward-facing GLUT3 conformation; G3iC, G3iE, and G3iF are inhibitors produced by TBVS of the inward-facing GLUT3 conformation.

### Effect of GLUT3 inhibitors on the transport activities of GLUT1, GLUT2, GLUT4, and GLUT5

**Figure 3:**
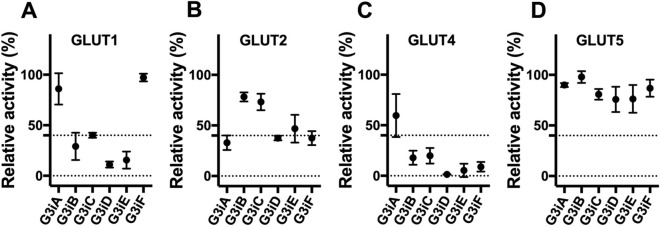
Effect of GLUT3 inhibitors on GLUT1, GLUT2, GLUT4, and GLUT5. The transport activity was assayed as the substrate uptake in hexose transporter-deficient yeast cells expressing functional GLUT1^65^, GLUT2^69^, GLUT4^65^, or GLUT5^66^. Substrate final concentration in the assay matched the corresponding substrate K_M_ and was 5 mM glucose for GLUT1 and GLUT4, 15 mM glucose for GLUT2_∆loopS_Q455R_, and 10 mM fructose for GLUT5_S72Y_. Transport assay was initiated by the addition of C^14^-labeled substrate and stopped after 10 min (see “Materials and methods” for details). **(A–D)** Effect of G3iA-G3iF, at 50 µM concentration, on the relative transport activities of GLUT1 **(A)**, GLUT2 **(B)**, GLUT4 **(C)**, and GLUT5 **(D)**. Standard deviations for experimental points come from at least three independent measurements.

The most studied GLUTs are Class 1 GLUTs (GLUT1-4) and a Class 2 GLUT, GLUT5 (a fructose-only transporter). GLUT3 sequence homology and identity is 79% and 64%, 71% and 52%, 74% and 57%, and 60% and 38% with GLUT1, GLUT2, GLUT4, and GLUT5, respectively^61^. The *hxt*^*0*^ yeast cell systems expressing functional GLUT1-4^65,69^ and GLUT5^66^ allow us to determine the in vivo transport activity (substrate uptake by a GLUT into whole cells) for individual GLUTs and establish GLUT3 inhibitor selectivity for these transporters. The transport assay was performed as described for GLUT3, but at the substrate concentrations that correspond to the K_M_ for each GLUT (i.e., 5 mM glucose for GLUT1 and GLUT4^65^, 15 mM glucose for GLUT2_∆loopS_Q455R_^63^, and 10 mM fructose for GLUT5_S72Y_^66^). Given the higher sequence similarity within Class 1 GLUTs, we expected GLUT3 inhibitors to affect Class 1 GLUTs much more than GLUT5. Indeed, G3iA-G3iF reduced the transport activities of some of the other Class 1 GLUTs (Fig. 3A–C, Table 2, Supplementary Fig. S6). In contrast, none of the GLUT3 inhibitors altered significantly GLUT5 transport activity (Fig. 3D). G3iA, the most potent GLUT3 inhibitor, showed the highest selectivity for GLUT3, inhibiting only GLUT2 but with ~ fourfold weaker potency (Table 2). Some GLUT3 inhibitors exhibited comparable inhibition for other Class 1 GLUTs. For example, G3iB was an equipotent inhibitor of GLUT4, while G3iD and G3iE inhibited GLUT1 in a similar manner to GLUT3. Surprisingly, two GLUT3 inhibitors, G3iD and G3iF, had ~ fivefold higher potency for GLUT4 than GLUT3 (Table 2). Moreover, G3iD affected all Class 1 GLUTs, having the best relative selectivity for GLUT4. Since G3iD was most effective against GLUT4, we checked its inhibition mode for this transporter. Dixon plot indicated that G3iD is a competitive inhibitor of GLUT4 glucose transport activity, with K_i_ = 2.65 ± 0.24 µM (Supplementary Fig. S7A).

**Table 2:** GLUT3 inhibitor selectivity.

IC_50_ (µM)						
	G3iA	G3iB	G3iC	G3iD	G3iE	G3iF
GLUT1	> 50	25.0 ± 8.6	49.4 ± 6.7	17.7 ± 2.3	22.4 ± 3.7	> 50
GLUT2	29.4 ± 6.3	> 50	> 50	40.0 ± 3.7	> 50	24.7 ± 8.4
GLUT3	7.01 ± 1.02	12.4 ± 4.0	12.5 ± 4.4	19.3 ± 4.7	23.6 ± 1.9	38.4 ± 11.8
GLUT4	> 50	12.7 ± 2.3	25.6 ± 6.6	3.88 ± 0.57	12.4 ± 2.5	6.94 ± 1.37
GLUT5	> 50	> 50	> 50	> 50	> 50	> 50

IC_50_ of G3iA-G3iF for transport inhibition of GLUT1-5 assayed in the *hxt*^*0*^ yeast assay systems. Dose response curves for GLUT3 are in Fig. 2, those for GLUT1, GLUT2, and GLUT4 are in the Supplementary Fig. S6. The effect of GLUT3 inhibitors on GLUT5 is shown in Fig. 3D.

### Effect of GLUT3 inhibitors on cancer cells viability

A possible therapeutic application of GLUT3 inhibitors is in oncology, especially for cancers in which GLUT3 is a poor prognosis marker. We tested the effect of GLUT3 inhibitors on the cell viability of several different cancer lines: myeloid leukemia (HL60 and U937), lung cancer (A549), colorectal adenocarcinoma (Caco-2), breast cancer (MCF7), hepatocellular carcinoma (HepG2), and choriocarcinoma (BeWo) cells. GLUT3 inhibitors, at five different concentrations, ranging from 2 to 100 µM, were incubated with cancer cells for 48 h. Then, cell viability was assessed with the MTT assay^74^. MTT data is shown in Supplementary Fig. S8 and summarized in Table 3. Among GLUT3 inhibitors, G3iA had the lowest impact on cell viability in all cancer cell lines, which suggests that none of the cancer cell lines tested relies solely on GLUT3 for glucose transport. The other GLUT3 inhibitors showed variable effect on cell viability, depending on the cell line (Table 3), probably reflecting different combinations of the expressed GLUTs^5^. G3iB and G3iE were most effective in reducing the survival of leukemia cell lines HL60 and U937 (CC_50_ < 20 µM). G3iC and G3iD also affected the leukemia cell lines but at double the concentrations of G3iB and G3iE. The next cancer cell line most impacted by G3iB–G3iE was lung cancer (A549 cells), with CC_50_ ranging from ~ 22 µM for G3iC to ~ 33 µM for G3iE. G3iB had some cytotoxic activity (CC_50_ < 50 µM) against the highest number of cancer cell lines (HL60, U937, A549, HepG2, and MCF7).

**Table 3:** CC_50_ of GLUT3 inhibitors in various cancer cell lines determined from MTT assays.

Cell line	CC_50_ (µM)					
	G3iA	G3iB	G3iC	G3iD	G3iE	G3iF
A549	> 100	23.2 ± 5.0	21.6 ± 2.5	32.5 ± 14.2	> 50	> 100
BeWo	> 100	> 50	> 100	> 100	> 50	~ 100
Caco2	> 100	> 50	> 100	> 100	> 50	> 100
HepG2	> 100	39.3 ± 4.0	> 100	> 100	> 100	> 100
HL60	> 100	17.7 ± 1.3	31.1 ± 3.8	28.7 ± 2.1	15.7 ± 0.8	> 50
U937	> 100	27.5 ± 1.9	> 50	> 50	~ 18	> 50
MCF7	> 100	42.6 ± 4.2	> 50	> 50	> 100	> 100

The concentration of compound that resulted in 50% reduction cell viability compared to control (i.e., 50% cytotoxic concentration or CC_50_) was determined for G3iA-G3iF. GLUT3 inhibitors at concentrations ranging from 2 to 100 µM were incubated for 48 h with leukemia cell lines (HL60, U937), A549 (lung cancer), BeWo (choriocarcinoma), MCF7 (breast cancer), HepG2 (liver cancer), and Caco-2 (colon cancer) cells. Cell viability was determined with the MTT assay (data in Supplementary Fig. S8). CC_50_ values were determined in GraphPad (https://www.graphpad.com).

### GLUT3 inhibitor docking from TBVS screening

**Figure 4:**
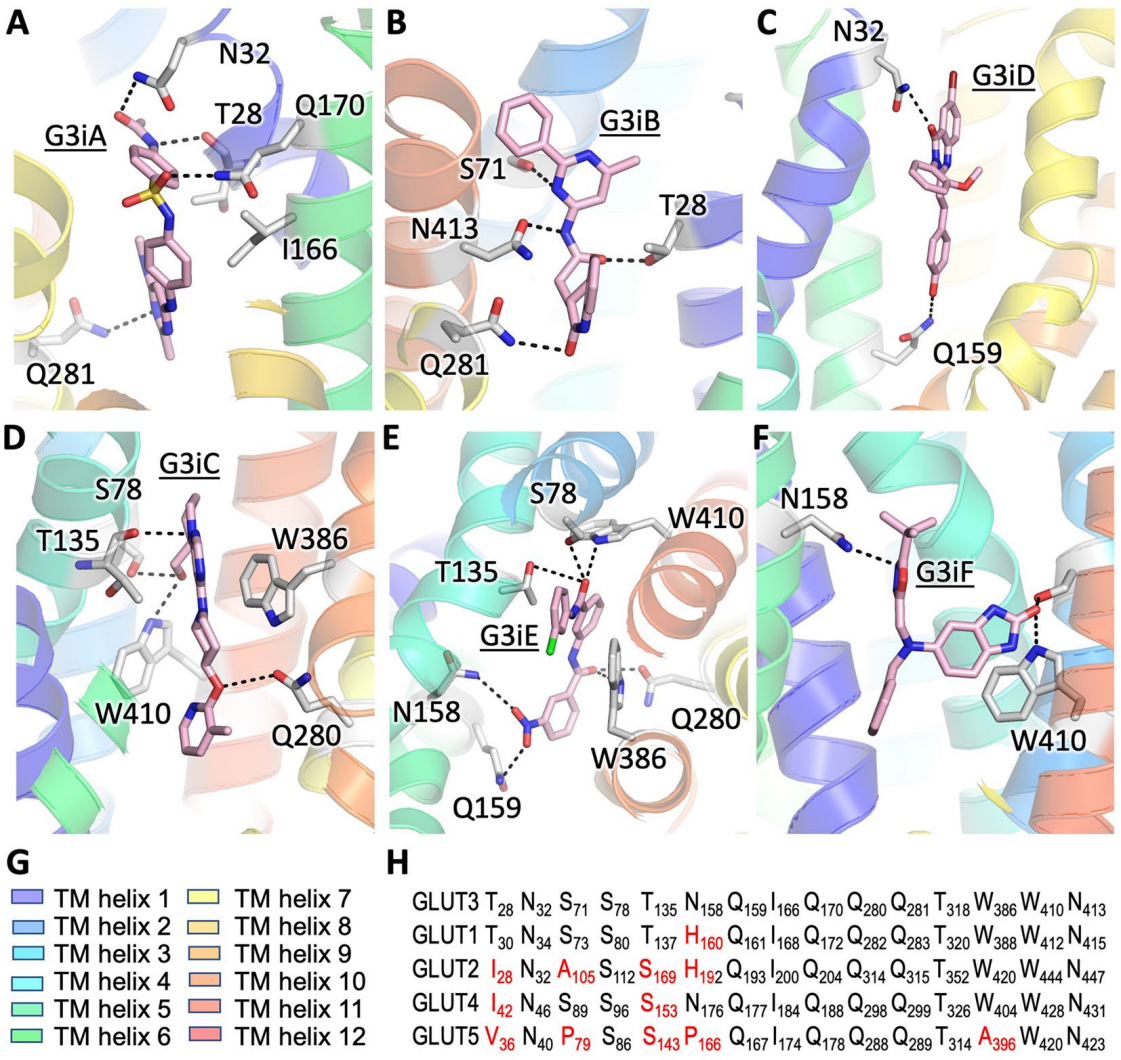
Docking sites of G3iA-G3iF in GLUT3 models. All GLUT3 inhibitors docked in the substrate binding site of the transporter, either to the outward-facing **(A–C)**, or inward-facing **(D-F)** conformations. GLUT3 inhibitors (pink color), G3iA **(A)**, G3iB **(B)**, G3iC **(D)**, G3iD **(C)**, G3iE **(E)**, and G3iF **(F)**, and the interacting protein residues (grey color) are shown as stick models. Dashed black lines indicate hydrogen-bond interactions. **(G)** Color-code for the transmembrane helices (same as in Fig. 1). **(H)** Sequence alignment among GLUT1-5, for the protein residues interacting with GLUT3 inhibitors. Substitutions of GLUT3 residues in the other GLUT sequences are indicated in red. The figures were generated with Pymol (https://www.pymol.org).

In our virtual ligand screening, all six compounds with GLUT3 inhibition activity docked to the glucose binding site of the transporter, in the outward-facing conformation (G3iA, G3iB, and G3iD) or the inward-facing one (G3iC, G3iE, and G3iF) (overview in Fig. 1, close-up views in Fig. 4, Supplementary Fig. S5, and Supplementary Fig. S9). Except for G3iE, all inhibitor docking sites fully or partially overlapped with the glucose binding site (Supplementary Fig. S5). Even for G3iE, the predicted docking site was very close to the active site, recruiting interactions with well-known glucose recognizing residues (Fig. 4E, Supplementary Fig. S5E).

G3iA has hydrogen bond interactions with the sidechains of N32, Q170, and Q281, and the backbone carbonyl of T28, and hydrophobic interactions with I166 (Fig. 4A). The hydrogen bonds of G3iB with protein sidechains involve residues T28, S71, N413, and Q281 (Fig. 4B). G3iD has hydrogen bonds with the carboxamide groups of N32 and Q159 (Fig. 4C). For the inward-facing conformation inhibitors, S78 and W410 are common to all the binding pockets. G3iC and G3iE also share T135, Q280, and W386 in their binding sites. N158 sidechain forms hydrogen bonds with both G3iE and G3iF, while Q159 interacts only with G3iE (Fig. 4D–F). Analysis of the sequence conservation among GLUT1-5 shows that most residues that interact with the GLUT3 inhibitors are conserved between GLUT1 and GLUT3, with N158 being the exception (Fig. 4H). T28 is replaced by a hydrophobic residue in GLUT2, 4, and 5. N158 is conserved in GLUT4; it is substituted conservatively with a histidine in GLUT1 and GLUT2 (H160 in GLUT1, H192 in GLUT2), but drastically with a proline in GLUT5 (P166). Docking G3iD to a model for the outward-facing conformation of GLUT4 (Supplementary Fig. S7B, Supplementary Fig. S10B), with the same ligand docking protocol as for the GLUT3 model, shows hydrogen bond interactions with T326 and N431 instead of N32 and Q159 of GLUT3 (Fig. 4C, Supplementary Fig. S10A). T326 and N431 are located in the TM helices 8 and 11, whereas N32 and Q159 are in TM helices 1 and 5. Thus, our modeling suggests that G3iD binds to the C-half of GLUT4, but to the N-half of GLUT3. Nevertheless, the predicted site still coincides in part with the glucose binding site (Supplementary Fig. S7B), as supported by the competitive inhibition mode of G3iD for GLUT4 (K_i_ = 2.65 ± 0.24 µM, Supplementary Fig. S7A). Docking of G3iF to GLUT4 (Supplementary Fig. S10D) also showed the relocation of the ligand binding site toward the C-half of GLUT4. G3iF binding site is made up of S96 (N-half residue) and three amino acids from the C-half (W404, N431 and W428) compared to GLUT3 (two N-half residues S78 and N158, and one C-half residue W410; Fig. 4F, Supplementary Fig. S10C). Hence, despite the residue conservation among Class 1 GLUTs in the predicted ligand binding sites of G3iA-G3iF in the GLUT3 structural models, we anticipate that molecular interactions of G3iA–G3iF compounds with the other transporters may vary. Comparably, the most altered ligand-transporter interaction is with GLUT5, given the radical residue substitutions in the predicted ligand sites for GLUT3, e.g., S71, N158, and W386 of GLUT3 are P79, P166, and A396 in GLUT5, respectively (Fig. 4H). In particular, the substitution of the bulky W386 with A395 shapes the substrate binding site affecting substrate specificity and inhibitor binding^75^.

## Discussion

By combining target-based virtual screening using the GLUT3 inward- and outward-facing conformations with the *hxt*^*0*^ yeast assays for individual GLUTs, we identified six new GLUT3 inhibitors. From the virtual screening, the 100 top-ranked candidates were selected for each GLUT3 conformation, for a total of 193 tested compounds. These compounds were ranked by the FLAP-calculated global similarity (GP) score after docking each compound within the glucose binding site of both GLUT3 conformations. We discovered three inhibitors for each transporter conformation (G3iA, G3iB, and G3iD are ligands for the outward-facing conformations; G3iC, G3iE and G3iF are ligands for the inward-facing conformation). Their predicted docking sites occupy (G3iA, G3iB, G3iC, G3iD, and G3iF) or are very close (G3iE) to the glucose binding site (Supplementary Fig. S5). The outward-facing conformation inhibitors probably inhibit the transport by directly hindering glucose access to its active site, as suggested by the ligand docking results (Fig. 4, Supplementary Fig. S5) and the competitive inhibition mode of G2iA (Supplementary Fig. S4A). The inward-facing conformation inhibitors may inhibit the transport by stabilizing the inward-facing conformation and, thus, preventing glucose from entering its active site in either conformation, accordant with the competitive inhibition mode determined for G3iC and G3iE (Supplementary Fig. S4B,C, Supplementary Fig. S5).

None of the six GLUT3 inhibitors showed activity against GLUT5 (a Class 2 GLUT). The most potent GLUT3 inhibitor, G3iA, appears relatively selective for GLUT3, compared with the other Class 1 GLUTs; it only affected GLUT2 with an IC_50_ fourfold higher than in GLUT3 (Table 2). The predicted G3iA docking site partially overlaps with glucose binding site (Supplementary Fig. S5A) consistent with the competitive inhibition mode for G3iA in GLUT3 (K_i_ ~ 3.5 µM, Supplementary Fig. S4A). Given its relative selectivity for GLUT3, G3iA may be uniquely suited to interfere with GLUT3 in glioblastoma, one of the most aggressive and lethal cancers where GLUT3 plays a central role. Indeed, the work of Libby et al.^76^ showed that GLUT3 inhibitors are effective in thwarting the growth of glioblastoma cells.

The rest of the GLUT3 inhibitors showed different levels of inhibition for the other Class 1 GLUTs. Interestingly, G3iD and G3iF inhibited GLUT4 more potently than GLUT3 (Table 2), with G3iD exhibiting relative selectivity for GLUT4 (IC_50_ ~ 3.9 µM, K_i_ ~ 2.7 µM) compared with the other GLUTs tested. Thus, G3iD joins other GLUT4-selective inhibitors^49^ that can be developed into drugs against multiple myeloma^48^ and other GLUT4-dependent cancers. G3iD and G3iE had comparable inhibition for GLUT1 and GLUT3, while G3iB affected similarly GLUT3 and GLUT4.

In several cancers, GLUT3 and GLUT1 upregulation cooccurs endowing cells with more flexibility in satisfying their high glucose demands. In these cancers, inhibitors that affect both GLUT1 and GLUT3, like G3iB, G3iD, and G3iE, might impair cancer cell survival better than GLUT-specific inhibitors. Indeed, combined loss of both GLUT1 and GLUT3 in lung adenocarcinoma mouse models significantly hindered tumor growth, whereas deletion of either GLUT isoform alone did not^77^. Cell viability assays in several cancer cell lines confirm this observation, with less specific GLUT3 inhibitors (G3iB-G3iE) having better cytotoxic activities than G3iA (Table 3). Among the cancer lines tested, leukemia and lung cancer cell lines were most affected by GLUT3 inhibitors G3iB-G3iE, pointing to a more significant reliance of these cells on glucose transport by Class 1 GLUTs. The GLUT3 inhibitor with cytotoxic activity for the highest number of cell lines was G3iB (Table 3). Interestingly, G3iB and G3iE had similar effects on the viability of leukemic cell lines HL60 and U937 (CC_50_ 16–20 µM) and shared comparable inhibitory actions on GLUT1 and GLUT4 (Table 2). Tilekar et al*.*^78,79^ found that compounds that inhibited both GLUT1 and GLUT4 hindered proliferation of CEM leukemia cells, both in vitro and in vivo. Thus, dual GLUT1 and GLUT4 inhibitors may have antileukemic potential. Further studies are needed to explore the relationship between flexibility of glucose transport in cancer cells through GLUT isoforms expression and GLUT inhibitor selectivity.

Although GLUT inhibitors with broader action may be more efficient in curtailing cancer cell survival, they may also be more cytotoxic for normal cells. On the other hand, inhibitors selective for one or two GLUT isoforms that predominate in certain cancers, when coupled with other anti-cancer treatments, may provide new therapeutic strategies that adjust depending on the metabolic reprogramming of the cancer type and stage.

The promiscuity of most GLUT3 inhibitors for the other Class 1 GLUTs may be due to the fact that we docked the ligands to the substrate binding site, in both transporter conformations, since the residues that form the glucose binding site in GLUT1-4 are fairly conserved in Class 1 GLUTs. Recent work by Schmidl et al*.*^63^ showed that ligands targeting the substrate cavity entrance, which is less conserved than the substrate binding site in Class 1 GLUTs, led to a high prevalence of GLUT2-specific inhibitors. Nevertheless, targeting the substrate binding site in virtual ligand screening does produce inhibitors that are Class-specific as illustrated here by GLUT3 inhibitors, which leave GLUT5 unaffected, or GLUT5 inhibitor MSNBA^62^, which does not inhibit Class 1 GLUTs and binds in the substrate site of the GLUT5 inward-facing conformation. A key difference in the substrate binding site between Class 1 and Class 2 GLUTs is W388 of GLUT1 (W386 of GLUT3), which is replaced with a smaller sidechain in Class 2 GLUTs, e.g., A396 of GLUT5. The sidechain in this position reshapes the substrate binding site influencing how inhibitors dock and substrate specificity^75^. This substitution, along with other radical differences in the predicted binding sites for GLUT3 inhibitors (e.g., proline substitutions in GLUT5 for S71 and N158 of GLUT3, Fig. 4H), may account for GLUT5 insensitivity to GLUT3 inhibitors.

An intriguing finding is that G3iD and G3iF have fivefold higher affinity for GLUT4 than GLUT3, showing preference for GLUT4 relative to the other Class 1 GLUTs (Table 2). Ligand docking of G3iD and G3iF to GLUT4 models, using the same protocol as for ligand docking to GLUT3 models, indicated that these inhibitors adopt different binding modes in the two transporters (Supplementary Fig. S10). Despite the distinct binding poses of G3iD in GLUT3 and GLUT4, the predicted docking site for G3iD in GLUT4 overlaps partly with the glucose binding site (Supplementary Fig. S7B), accordant with the competitive inhibition mode of this inhibitor in GLUT4 (Supplementary Fig. S7A). Overall, G3iD and G3iF have more interactions with protein residues in GLUT4 compared with GLUT3, consistent with the difference in inhibition potency observed.

In conclusion, we described the discovery of six new GLUT3 inhibitors. We used an ad hoc structure-based virtual screening protocol and an experimental assay where GLUT3 is expressed in a hexose transporter-deficient yeast strain to select and validate the inhibitory behavior of 193 small molecules candidates, narrowed down from an initial 8-million compound library. The IC_50_ of the six active compounds ranged from 7 to 38 µM. Furthermore, their selectivity profile was measured across several GLUTs (all Class 1 GLUTs and GLUT5). One inhibitor (G3iA) appears to be selective against GLUT3. The other five inhibitors show different degrees of cross-reactivity with one or more Class 1 GLUTs. None of the six GLUT3 inhibitors blocked GLUT5 activity. The compounds identified in this study are promising candidates for the development of new anticancer drugs via GLUT3, GLUT1, and GLUT4 inhibition, as these inhibitors exhibit different selectivity and affinity for these three transporters, which are overexpressed in many cancers. In particular, the survival of leukemia and lung cancer cell lines is curbed by G3iB, G3iC, and G3iE. Our future plans include the identification and evaluation of similar compounds to improve their potency and selectivity.

## Materials and methods

Hexose transporter deficient yeast strains (*hxt*^*0*^) EBY.VW4000 for expression of GLUT5, EBY.S7 (carrying the *fgy1* mutation) for expression of GLUT1-3, and SDY.022 (EBY.S7 *Δerg4*) for expression of GLUT4 cells, and corresponding plasmids^65,66,73^ were from the labs of Drs. Eckhard Boles and Mislav Oreb (Goethe University, Germany). Media ingredients and buffer components were from VWR or Millipore Sigma. Commercial providers for chemicals tested for GLUT3 inhibition are listed in Supplementary Table S1. C^14^-glucose was from Moravek Inc (Brea, CA, USA). Cancer cell lines were from ATCC (https://www.atcc.org).

### Target-based virtual ligand screening procedure

The 3D structure of GLUT3, in the outward open conformation, was downloaded from the Protein Data Bank (PDB: 5C65). At the time, no crystal structure depicting the protein in an inward open conformation was available. Hence, a homology model was built starting from the GLUT3 amino acids sequence, with the inward open 3D structure of GLUT1 (PDB: 4PYP) as the template. The software MOE (https://www.chemcomp.com/) was employed for generating the structural model, keeping the default calculation parameters. The same procedures were applied for the generation of the GLUT4 inward-facing and outward facing conformation models, using as templates PDB ID 4PYP and PDB ID 5C65, respectively. The extraction of the main binding site for both conformations and the following similarity-based virtual screening were conducted by means of the Fingerprints for Ligands And Proteins (FLAP) program^72^. FLAP uses the GRID molecular interaction fields (MIFs)^80^ for estimating the similarity between a template (usually an active compound or a binding site) and a collection of molecules to be screened. In this study, the ChemNavigator collection (Millipore Sigma) of around 8 million molecules was screened. For each virtually screened molecule, the chemical structure was normalized in terms of pKa by generating the most abundant form in water at pH 7.4 with the software MoKa^81^. When importing a molecule, FLAP automatically generates 3D coordinates, a certain number of conformers (up to 100 in this case) and computes the MIFs for each of them. Four GRID probes are used with default settings for computing MIFs. The H probe maps the size and shape of the molecule, and the other 3 probes that map the interaction areas around the molecule: respectively, the N1, O and DRY probes map the hydrogen bond acceptor, the hydrogen bond donor, and the hydrophobic interaction regions. MIFs are then saved as 3D geometrical entities called quadruplets, which are formed by four relevant points extracted from the molecule MIFs that are connected among each other. Depending on the molecule, a different number of quadruplets is extracted. Finally, the alignment to the template (active ligand or pocket) is performed by overlapping the quadruplets of each conformer with the quadruples extracted from the template. Once the alignment is complete, similarity scores are computed from the original MIFs of the screened molecule and template. Amongst various similarity scores computed by FLAP, the Glob-Prod (GP) score measures the overall GRID fields similarity between the template and the screened molecule. With this procedure, all 8 million molecules were screened with the pockets extracted from both the inward and outward GLUT3 structures and ranked according to the computed GP values. Finally, the top 100 molecules, having the highest GP values, were selected from each pocket, for a total of 200 molecules to be tested in inhibition experiments in vitro.

### Culturing of GLUT-expressing *hxt*^*0*^ yeast cells for transport assay

Depending on the plasmid selection marker, the media for cell culturing was either YEP [1% (w/v) yeast extract and 2% (w/v) peptone] or complete synthetic media without uracil (SC-uracil). Yeast cell culturing was done at 30 ºC with shaking (180–220 rpm).

The plasmids containing functional constructs of GLUT1-5 were transformed in the corresponding *hxt*^*0*^ strains^65,66,73^ (EBY.VW4000 for GLUT5_S72Y_, EBY.S7 for GLUT1, GLUT2_∆loopS_Q455R_, GLUT3 and GLUT3_S66Y_, and SDY.022 for GLUT4) and grown on 2% (w/v) agar plates of the respective media supplemented with 1% (w/v) maltose. An initial culture of ~ 10 ml was started with a few colonies and grown for 2–3 days if the media was SC-uracil with 1% (w/v) maltose (GLUT1, GLUT3, GLUT4) or 1–2 days if the media was YEP with 1% (w/v) maltose and 100 µg/ml geneticin G418 (GLUT2_∆loopS_Q455R_ and GLUT5_S72Y_). Cells were washed once in the corresponding media in which maltose was substituted with 0.1–2% hexose substrate for the expressing GLUT (i.e., SC-uracil, 2% (w/v) glucose for GLUT1; SC-uracil, 0.2% (w/v/) glucose for GLUT3 and GLUT4; YEP, 0.2% (w/v) glucose, 100 µg/ml geneticin G418 for GLUT2_∆loopS_Q455R_, and YEP, 2% (w/v) fructose for GLUT5 _S72Y_). Then, cells were transferred in the same media so that OD_600nm_ ~ 0.5 and grown further for 1–2 days.

### GLUT transport assay

For transport activity assay, cells in the hexose media were centrifuged (1000×*g*, 5 min, room temperature), washed once with PBS buffer (10 mM Na_2_HPO_4_, 1.8 mM KH_2_PO_4_, 2.7 mM KCl, 137 mM NaCl, pH 7.4), and resuspended in PBS buffer at an OD_600nm_ ~ 10; each assay contained 100 µl of this cell solution. Transport activity assay was started by adding C^14^-hexose at a substrate concentration corresponding to the respective K_M_ (5 mM glucose for GLUT1 or GLUT4, 3 mM glucose for GLUT3 or 1.5 mM glucose for GLUT3_S66Y_, 15 mM glucose for GLUT2_∆loopS_Q455R_, and 10 mM fructose for GLUT5_S72Y_); for determining the glucose K_M_ in GLUT3 and GLUT3_S66Y_, substrate concentrations were varied accordingly. Substrate uptake into cells was linear for more than 20 min. Transport activity was stopped after 10 min by adding 3-ml ice-chilled Quench buffer (0.1 M KPi, 0.1 M LiCl, pH 5.5), followed by filtration through a glass fiber channel (GC50; Advantec, Tokyo, Japan) under vacuum, and another wash with 3-ml Quench buffer and filtration. The filtration membranes were transferred into scintillation vials with 10 ml of Scintillation Solution (BioSafeII; Research Products International, Mount Prospect, IL, USA), and vortexed briefly. The radioactivity was determined with a scintillation counter (Tri-carb 2900TR, Perkin Elmer, USA). As all compounds were solubilized in dimethyl sulfoxide (DMSO), controls for determining the relative transport activity included 1% (v/v) DMSO to represent DMSO used as vehicle control (i.e., 100% activity), and known inhibitors at saturating concentrations, 200 µM phloretin for GLUT1-4^69,82^, and 100 µM N-[4-(methylsulfonyl)-2-nitrophenyl]-1,3-benzodioxol-5-amine (MSNBA) for GLUT5 (i.e., complete inhibition). Primary screening was done at 100 µM compound concentration (see Table S1 for a list of all tested compounds), and the IC_50_ values were further calculated for the compounds that diminished the relative transport activity by at least 60%. Data were analyzed with GraphPad Prism (San Diego, CA, USA).

### MTT cancer cell viability assay in the presence of GLUT3 inhibitors

Cancer cell viability in the presence of GLUT3 inhibitors was assayed using the MTT Cell Viability Kit (Biotium Inc, Freemont, CA) according to manufacturer’s instructions. Cancer cells were grown in the corresponding medium, at 37 °C and 5% CO_2_ in a humidified incubator. The media (Gibco) for leukemia cells (HL60 and U937), A549, MCF7, BeWo, and HepG2 cells were RPMI 1640, Ham’s F-12K, DMEM:F-12, Ham’s F-12K, and EMEM, respectively, all supplemented with 10% (v/v) fetal bovine serum (HyClone). Caco-2 cells were grown in EMEM supplemented with 20% (v/v) fetal bovine serum. Cells were detached from the culture flasks with Accutase (Innovative Cell Technologies, Inc., San Diego, CA) according to manufacturer’s instructions. Cells were seeded in 96-well plates, at a density of ~ 30,000 cells/well (in 100 µl fresh media) and incubated with varying concentrations of GLUT3 inhibitors for 48 h, at 37 °C and 5% CO_2_ in a humidified incubator. The inhibitors were dissolved in DMSO, at 100× the final concentration, so that 1 µl of the stock inhibitor concentration was added per well, making the final DMSO concentration 1% (v/v). Therefore, controls for 100% cell viability were cells with 1% (v/v) DMSO without the inhibitors. Controls for 0% cell viability were cells treated with 0.3% (v/v) Tween 20. For MTT assay, 10 µl of MTT reagent was added in each well followed by 3–4 h incubation at 37 °C and 5% CO2 in a humidified incubator. After thoroughly mixing in 200 µl DMSO in each well, the plates were put into a plate reader (BioTek Synergy H1), and the absorbances at 570 nm and 630 nm were measured with Gen5 program (BioTek Inc.). The experimental values represent Abs_570 nm_-Abs_630 nm_ with each inhibitor condition and control being the average of three wells.

## Supplementary Information

Supplementary Information.
