# Supplementary material for: GLUT3 inhibitor discovery through in silico ligand screening and in vivo validation in eukaryotic expression systems
Source: Sci Rep. 2022 Jan 26;12:1429. doi: 10.1038/s41598-022-05383-9 (PMC8791944; doi:10.1038/s41598-022-05383-9)
Supplement: Supplementary file 1 — Supplementary Information. [file 41598_2022_5383_MOESM1_ESM.docx]

**SUPPLEMENTARY INFORMATION**

**GLUT3 inhibitor discovery through *in silico* ligand screening and *in vivo* validation in eukaryotic expression systems**

Cristina V. Iancu^1,#^, Giovanni Bocci^2,#^, Mohd Ishtikhar ^1,#^, Moumita Khamrai^1^, Mislav Oreb^3^, Tudor I. Oprea^2,4,5,6*^, and Jun-yong Choe^1,7,8*^

^1^ East Carolina Diabetes and Obesity Institute, East Carolina University, Greenville, NC 27834, USA.

^2^ Translational Informatics Division, Department of Internal Medicine, The University of New Mexico School of Medicine, Albuquerque, NM 87131, USA.

^3^ Institute of Molecular Biosciences, Faculty of Biological Sciences, Goethe University Frankfurt, Frankfurt am Main, Germany.

^4^ UNM Comprehensive Cancer Center, The University of New Mexico, Albuquerque, NM 87131, USA.

^5^ Department of Rheumatology and Inflammation Research, Institute of Medicine, Sahlgrenska Academy at University of Gothenburg, Gothenburg, Sweden.

^6^ Novo Nordisk Foundation Center for Protein Research, Faculty of Health and Medical Sciences, University of Copenhagen, Copenhagen, Denmark.

^7^ Department of Chemistry, East Carolina Diabetes and Obesity Institute, East Carolina University, Greenville, NC 27834, USA.

^8^ Department of Biochemistry and Molecular Biology, Rosalind Franklin University, North Chicago, IL 60064 USA.

^#^ Contributed equally

^*^ Corresponding authors: Tudor Oprea, toprea@salud.unm.edu

Jun-yong Choe, choej18@ecu.edu

Supplementary Figure S1

Supplementary Figure S2

Supplementary Figure S3

Supplementary Figure S4

Supplementary Figure S5

Supplementary Figure S6

Supplementary Figure S7

Supplementary Figure S8

Supplementary Figure S9

Supplementary Figure S10

Supplementary Table S1

Supplementary Table S2


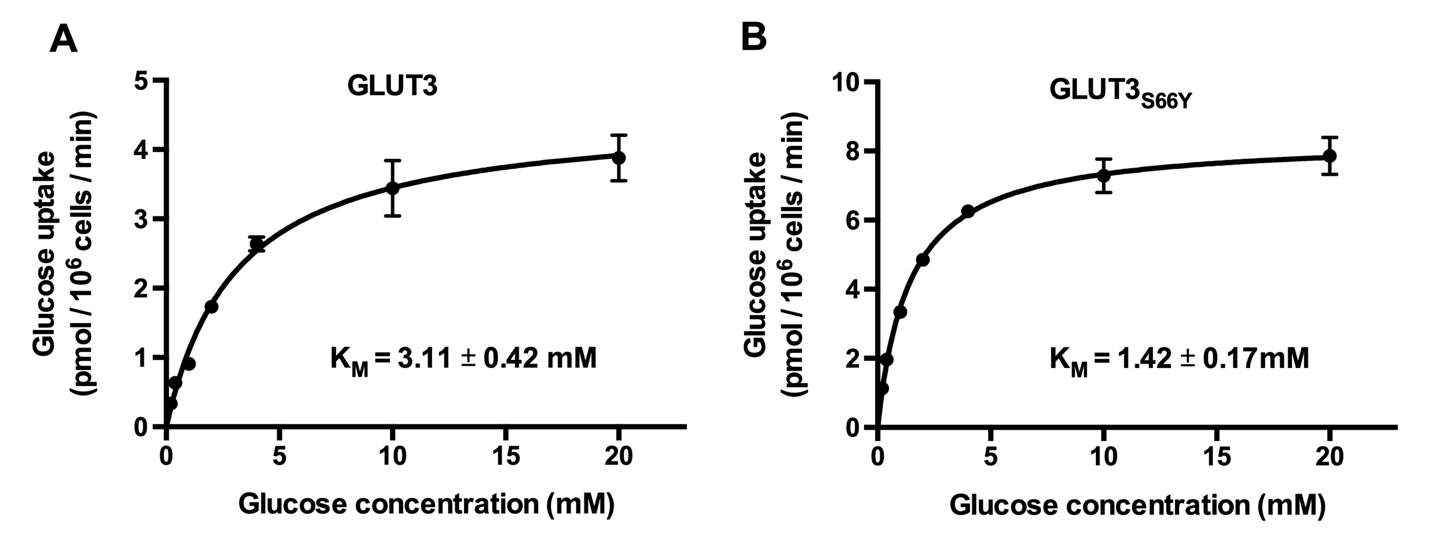


**Supplementary Figure S1. Michaelis-Menten curves for glucose transport in GLUT3 yeast systems.** Glucose transport activity of GLUT3 wild-type **(A)** or S66Y mutant **(B)** expressed in hexose transporter-deficient (*hxt^0^*) EBY.S7 yeast cells^1^ at different glucose concentrations. Transport activity was initiated by the addition of C^14^-glucose to the yeast cells in PBS buffer. After 10 mins, transport activity was stopped, and the radioactivity accumulated in cells was measured. See Materials and Methods for details. Error bars represent standard deviation of three independent measurements. Graphs and data analysis for K_M_ calculation were generated with GraphPad (www.graphpad.com).

**Supplementary Figure S2. Effect of top-ranked ligand candidates from TBVS on GLUT3 transport activity.** The tested compounds (listed in Supplementary Table S1) are identified by the ChemNavigator Structure ID. Relative transport activity of GLUT3 expressed in EBY.S7 yeast cells in the presence of 100 µM compound concentration, at 3 mM glucose concentration (i.e., glucose K_M_ concentration, see Supplementary Figure S1A). More details are in Materials and Methods. Error bars represent the standard deviation from three independent measurements.

**
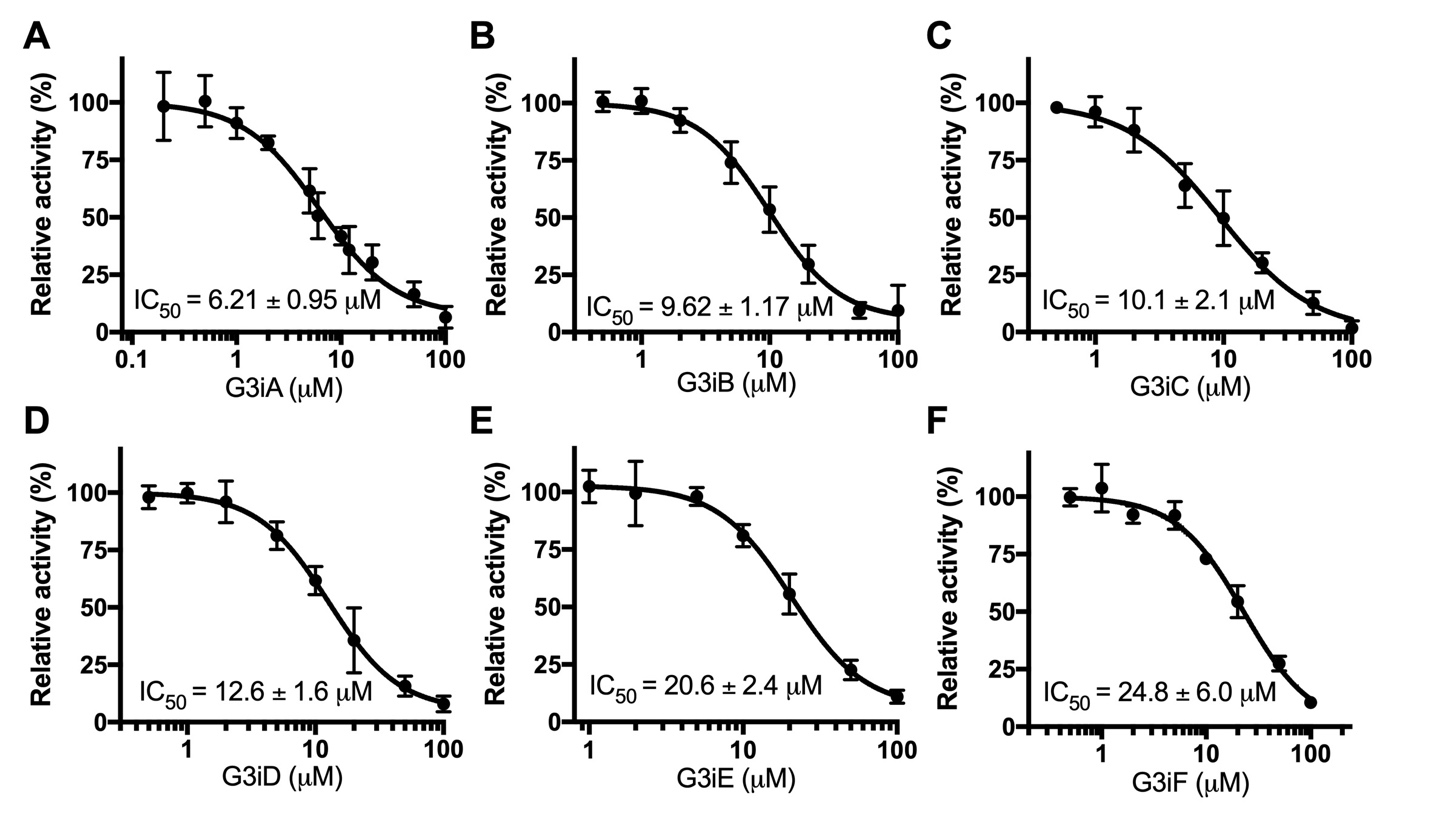
**

**Supplementary Figure S3. Dose response curves for G3iA-G3iF inhibition of GLUT3_S66Y_.** The relative glucose transport activity of GLUT3_S66Y_ in EBY.S7 *hxt^0^* yeast cells was assayed at 1.5 mM glucose concentration to match the glucose K_M_ (Supplementary Figure S1B). Error bars represent standard deviations from at least three independent measurements. Graphs, data analysis, and IC_50_ values were generated with GraphPad (www.graphpad.com).

**Supplementary Figure S4. Inhibition mode of G3iA, G3iC, and G3iE in GLUT3**. **(A-C)** Dixon plots^2^ for GLUT3_S66Y_ inhibition by G3iA, G3iC, and G3iE, respectively**.** The glucose transport activity of GLUT3_S66Y_ in EBY.S7 *hxt^0^* yeast cells was assayed at 0.75, 1.50, and 3.00 mM glucose concentrations, with different inhibitor concentrations. The radioisotope assay is described in Materials and Methods. Data analysis and the graphs were generated with GraphPad Prism (https://www.graphpad.com). All inhibitors showed competitive inhibition with glucose, with K_i,G3iA_ = 3.49 ± 0.47 µM **(A),** K_i,G3iC_ = 5.83 ± 0.30 µM **(B)** and K_i,G3iE_ = 12.3 ± 0.9 µM **(C)**. Error bars represent standard deviations from at least three independent measurements.


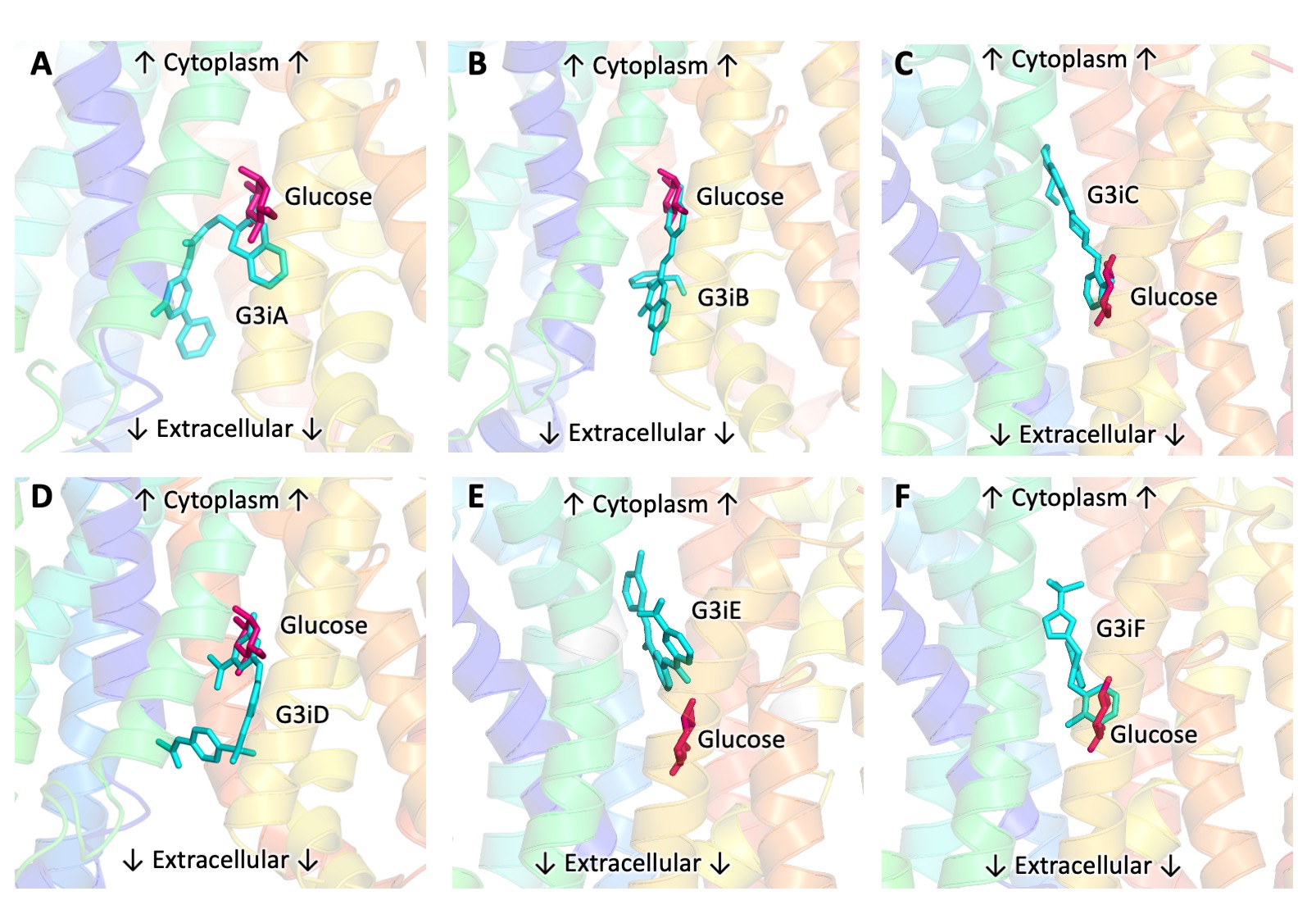


**Supplementary Figure S5. Predicted docking sites of GLUT3 inhibitors relative to the glucose binding site in the GLUT3 models.** Close-up of the glucose binding site in the outward-facing **(A, B, D)** and inward-facing **(C, E, F)** GLUT3 models**,** showing the predicted docking sites from TBVS for G3iA **(A),** G3iB **(B),** G3iC **(C),** G3iD **(D),** G3iE **(E)** and G3iF **(F)**. The structural models for the inward-facing **(A, B, D)** and outward-facing **(C, E, F)** conformations of GLUT3 were generated as described in the Material and Methods. GLUT3 inhibitors (cyan), and glucose modeled in its active site (red) are shown as stick models. Glucose modeling was based on the glucosyl moiety of nonyl beta-D-glucopyranoside from PDB ID 4PYP **(C, E, F)** and alpha-D-glucopyranose-(1-4)-alpha-D-glucopyranose from PDB ID 4ZWC **(A, B, D)**. Figures were generated with Pymol (https://www.pymol.org).

**Supplementary Figure S6. Dose response curves for GLUT3 inhibitors on the transport activities of GLUT1, GLUT2, and GLUT4 in *hxt^0^* yeast cells.** Dose response curves for GLUT3 inhibitors in GLUT1 (top row), GLUT2 (middle row), and GLUT4 (bottom row). Substrate conditions for relative transport activity matched the corresponding substrate K_M_: 15 mM glucose for GLUT2, and 5 mM glucose for GLUT1 and GLUT4. Glucose transport activity of functional GLUTs (GLUT1^3^, GLUT2_∆loopS_Q455R_^1^, and GLUT4^3^) expressed in *hxt^0^* yeast cells (EBY.S7 cells for GLUT1 and GLUT2_∆loopS_Q455R_, and SDY.022 cells for GLUT4) was measured as C^14^-glucose uptake in whole cells (see Materials and Methods for details). Error bars represent standard deviations from at least three independent measurements. Graphs, data analysis, and IC_50_ values were generated with GraphPad (www.graphpad.com).

**Supplementary Figure S7. G3iD inhibition in GLUT4**. **(A)** Dixon plot^2^ for GLUT4 inhibition by G3iD. The glucose transport activity of GLUT4 in SDY.022 *hxt^0^* yeast cells was assayed at 2.5, 5, and 10 mM glucose concentrations, in the presence of 0, 4, and 8 µM G3iD concentration. The radioisotope assay is described in Materials and Methods. Data analysis and the graph were generated with GraphPad Prism (https://www.graphpad.com). G3iD was competitive with glucose, with K_i,G3iD_ = 2.65 ± 0.24 µM. Error bars represent standard deviations from at least three independent measurements. **(B)** Close-up of the glucose binding site in the outward-facing model of GLUT4, showing the predicted docking site for G3iD. Glucose (cyan), G3iD (pink), and protein residues interacting with the inhibitor (grey) are shown as stick models. The structural model for GLUT4 was based on the GLUT1 crystal structure PDB ID 4PYP and generated as described in the Material and Methods. The figure was generated with Pymol (https://www.pymol.org).

**
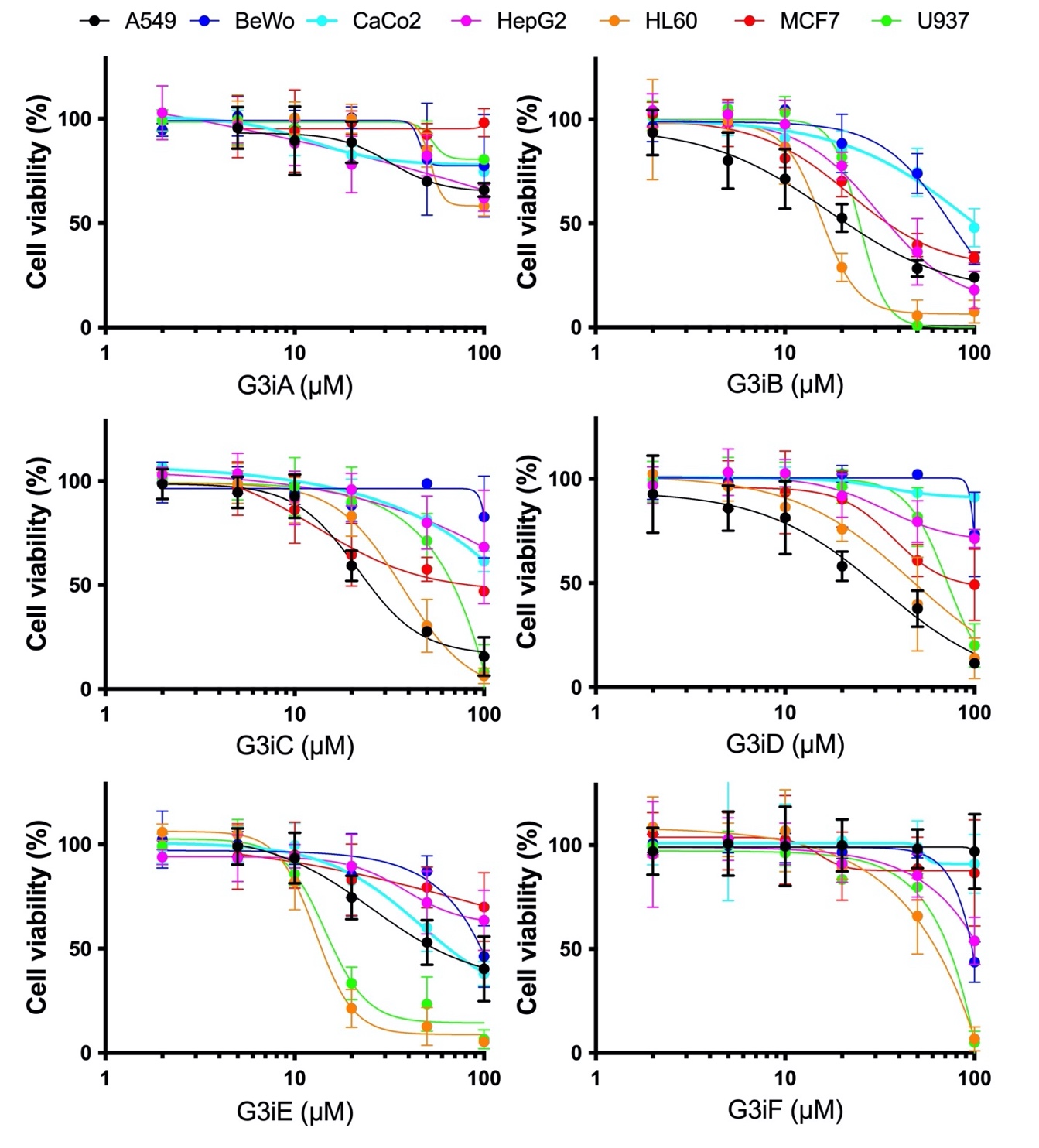
**

**Supplementary Figure S8. Effect of GLUT3 inhibitors on the viability of various cancer cell lines.** Cell viability was determined by MTT assay in A549 (lung cancer), BeWo (choriocarcinoma), Caco-2 (colon cancer), HepG2 (liver cancer), HL-60 (leukemia), MCF7 (breast cancer), and U937 (histiocytic lymphoma) cell lines. GLUT3 inhibitors at concentrations of 2, 5, 10, 20, 50, and 100 µM were incubated with cells in 96-well plates for 48 hours before the addition of the MTT reagent.

A. G3iA B. G3iB


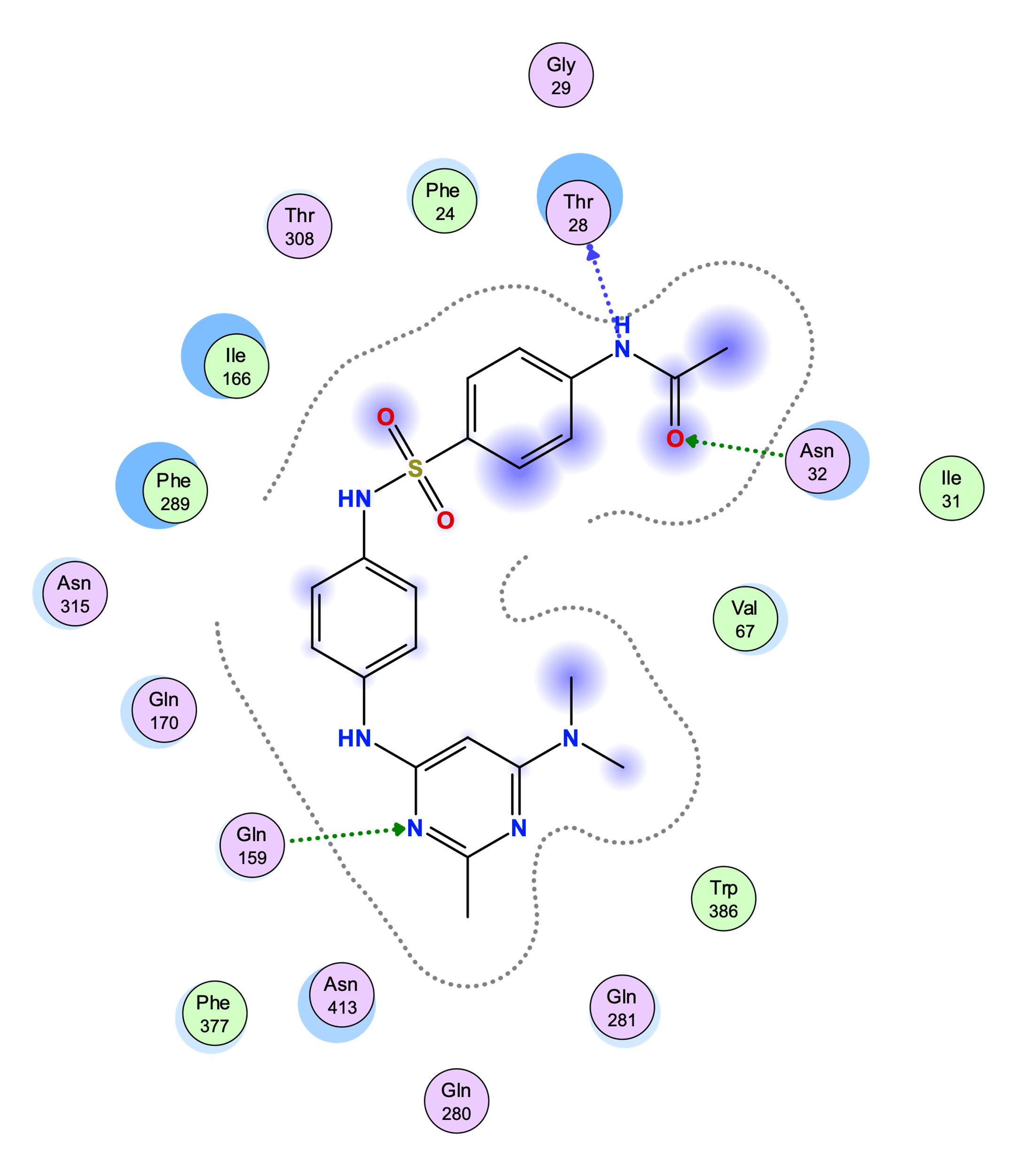

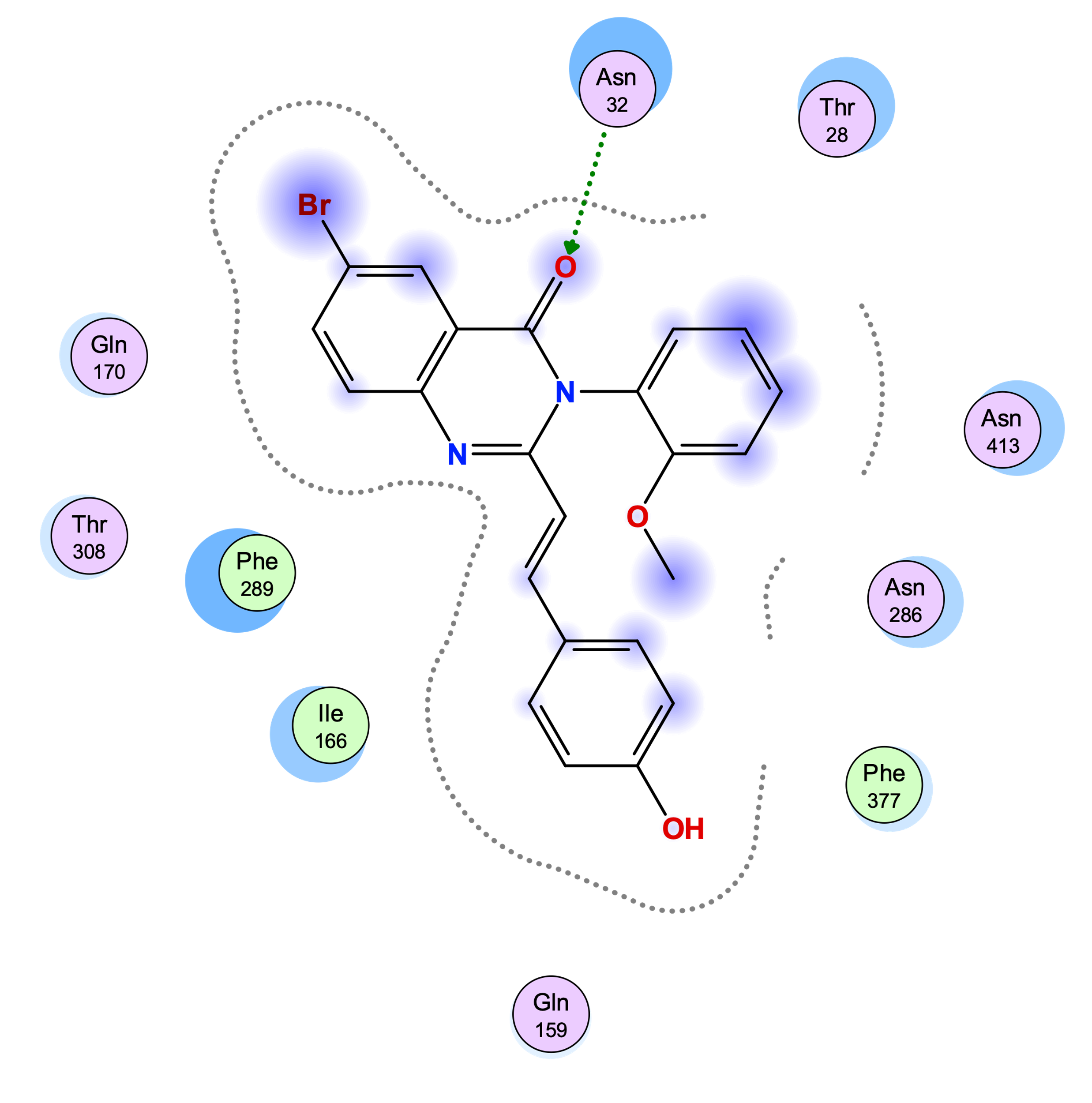


C. G3iC D. G3iD


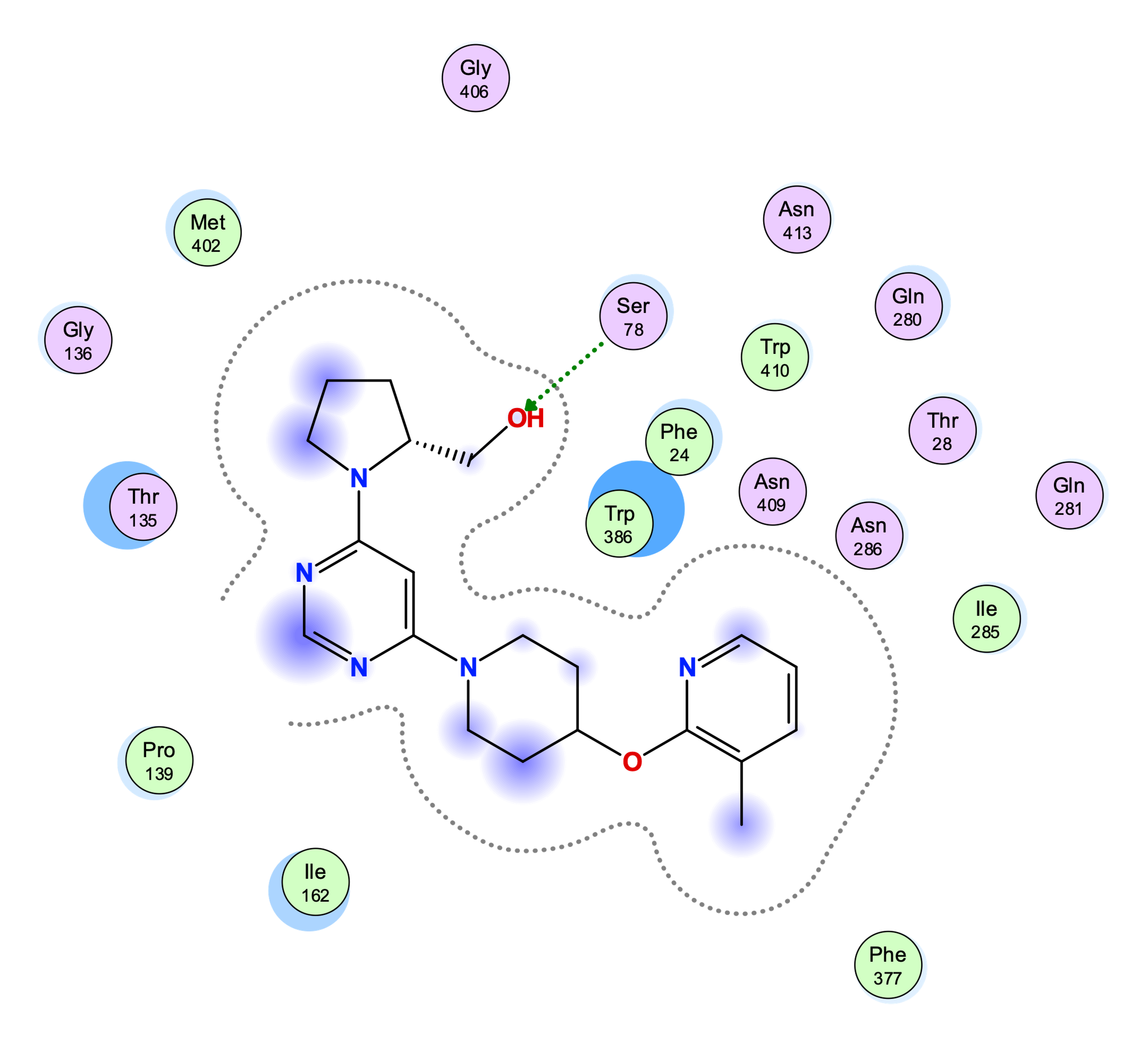

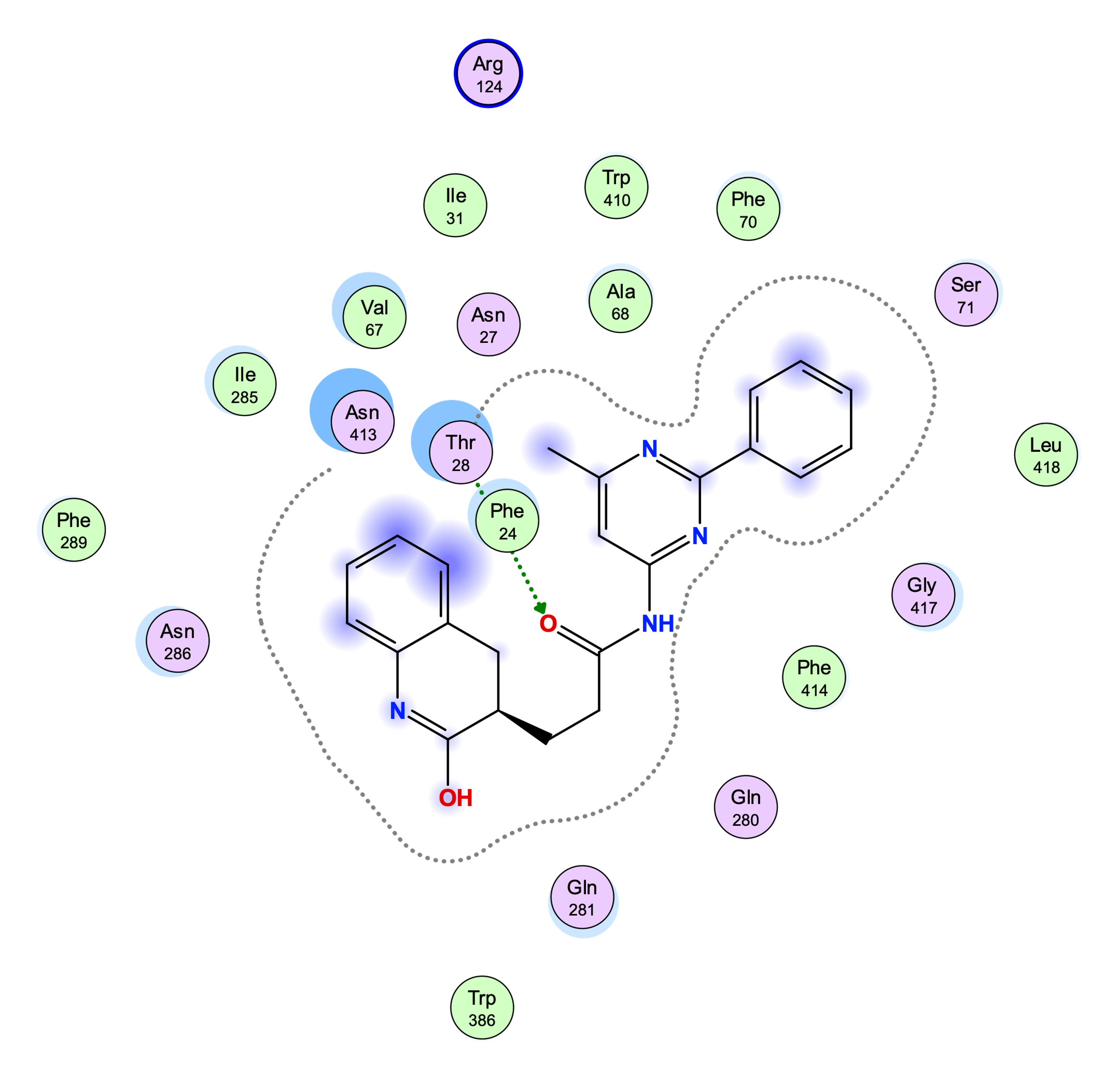


E. G3iE F. G3iF


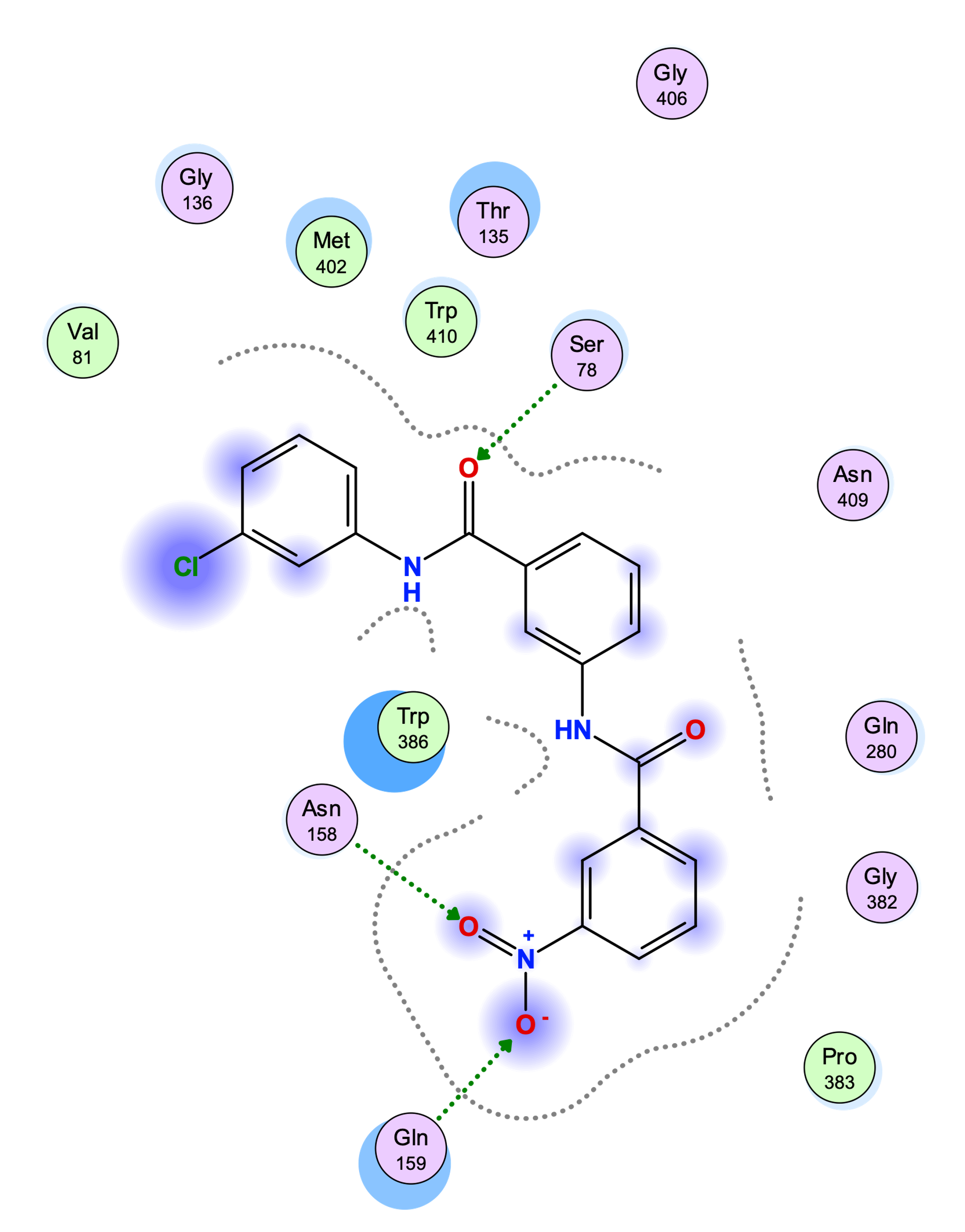

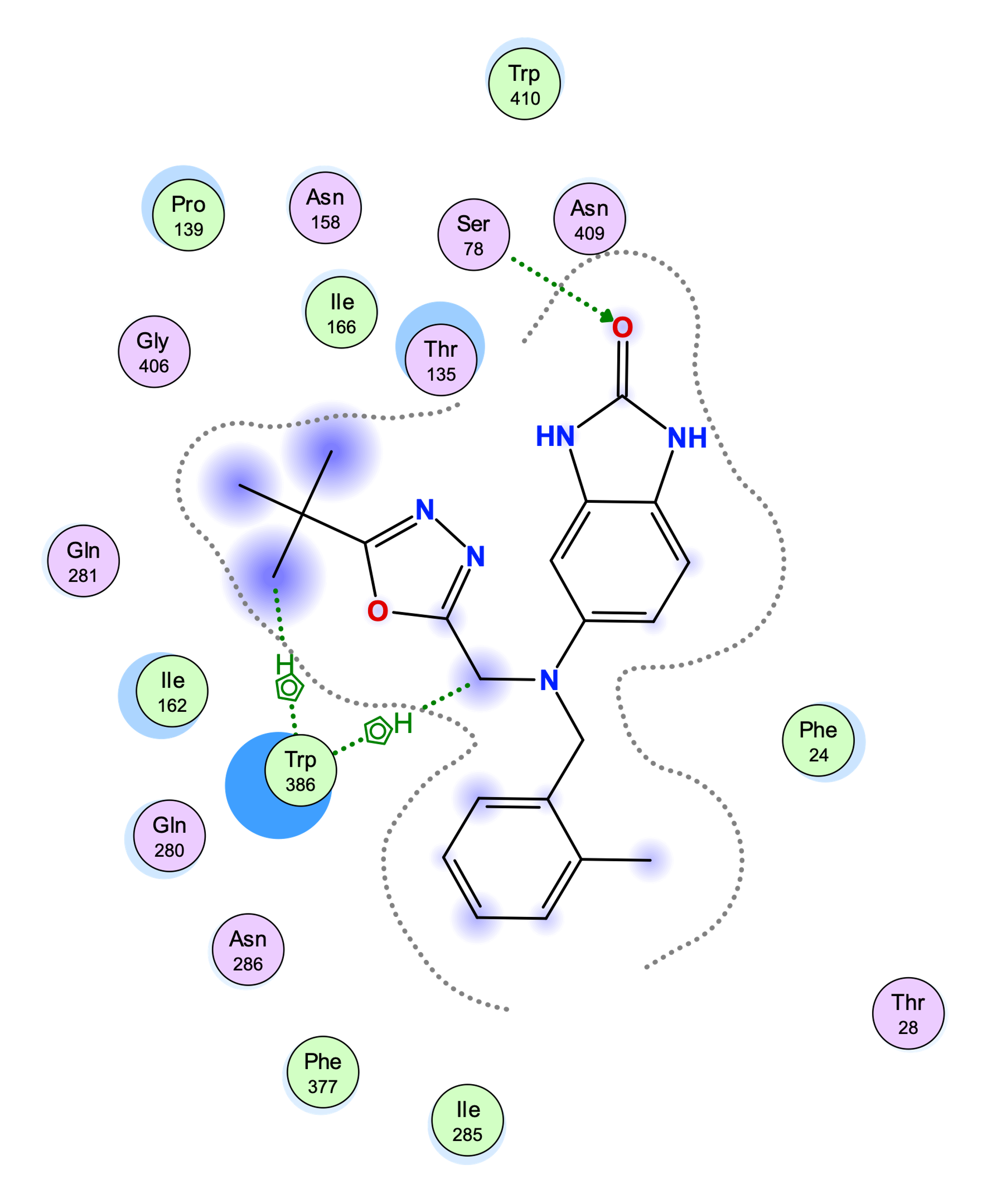


G. Legend


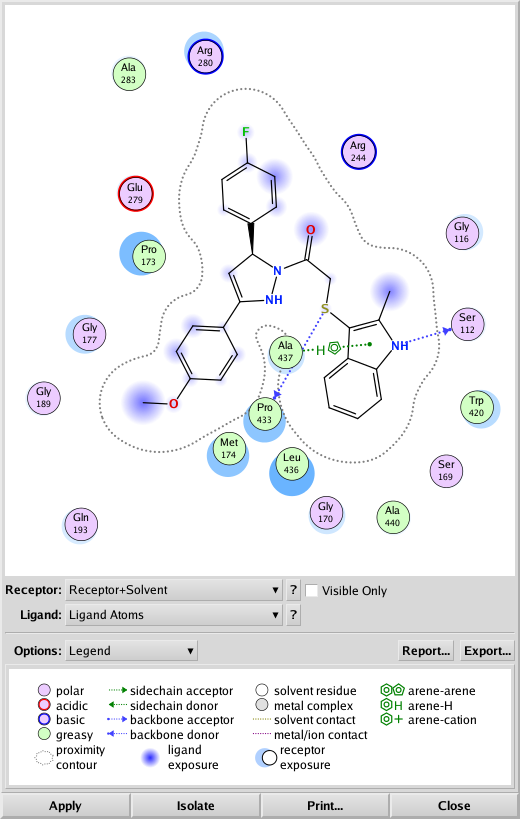


**Supplementary Figure S9. Protein-ligand interactions for docked GLUT3 inhibitors to the outward-facing conformation GLUT3 model (A, B, D) and inward-facing conformation GLUT3 model (C, E, F).** The ligand-protein interaction diagrams for G3iA **(A)**, G3iB **(B)**, G3iC **(C)**, G3iD **(D)**, G3iE **(E)**, and G3iF **(F)**, were generated in MOE (https://www.chemcomp.com) with the program “Ligand Interactions”. **(G)** Legend for the type of residues and ligand-protein interactions in **(A-F)**.

**Supplementary Figure S10. Docking of G3iD and G3iF to GLUT3 and GLUT4 models.** The homology models for GLUT3 and GLUT4 were generated in MOE using as templates the crystal structure of GLUT1 (PDB ID 4PYP) for the inward-facing conformations, and the GLUT3 crystal structure (PDB ID 5C65) for the outward-facing. Views from the extracellular side of the transporter showing G3iD docked to the outward-facing conformation of GLUT3 **(A)** or GLUT4 **(B)**. Views from the cytoplasmic side of G3iF docked to the inward-facing conformation of GLUT3 **(C)** and GLUT4 **(D)**. The transmembrane helices are denoted as H1-12, and their color code is as in Figure 4G. Helices H1-H6 belong to the N-half of the transporter, helices H7-H12 belong to the C-half of the transporter. The figures were generated with Pymol (https://www.pymol.org).

**Supplementary Table S1. Top-scored ligand candidates from target-based virtual screening that were purchased and tested for GLUT3 transport inhibition.** The ChemNavigator structure identifier (Structure_ID), commercial information (Supplier and Catalog #), chemical name, SMILES code, and molecular formula (MF) are indicated for each compound (except for some unavailable chemicals’ names). Identified GLUT3 inhibitors (G3iA-G3iF) are in red font.

| **CHEMICAL** | | | | **SUPPLIER** | |
| --- | --- | --- | --- | --- | --- |
| **Structure_ID** | **Name** | **SMILES** | **MF** | **Name** | **Catalog #** |
| 282359772 | 1-(1,3-benzoxazol-2-yl)-N-[2-(3-chlorophenyl)-2-hydroxyethyl]-4-piperidinecarboxamide | Clc1cc(ccc1)C(O)CNC(=O)C2CCN(CC2)c3nc4c([o]3)cccc4 | C21H22O3N3Cl1 | Enamine | Z384626092 |
| 410456137 | 1-(2-(3,5-dimethyl-1H-1,2,4-triazol-1-yl)phenyl)-3-((3-hydroxycyclohexyl)methyl)urea | [n]3(nc(nc3C)C)c1c(cccc1)NC(=O)NCC2CC(CCC2)O | C18H25O2N5 | Enamine | Z1531376630 |
| 926266993 |  | [n]21ncnc2CCC(C1)NC(=O)NCc3cc(c(cc3)c4ccccc4)C | C21H23O1N5 | Enamine | Z1752450232 |
| 369122635 | 2-(4-{[6-(cyclopropylmethoxy)-3-pyridinyl]carbonyl}-1-piperazinyl)cyclopentanol | N2(CCN(CC2)C(=O)c3cnc(cc3)OCC4CC4)C1C(CCC1)O | C19H27O3N3 | Enamine | Z1143005635 |
| 124031033 | 3-{[3-(2-oxo-1,2,3,4-tetrahydro-3-quinolinyl)propanoyl]amino}-4-(1-pyrrolidinyl)benzamide | N4(CCCC4)c1c(cc(cc1)C(=O)N)NC(=O)CCC2Cc3c(cccc3)NC2=O | C23H26O3N4 | Enamine | Z238859824 |
| 155978154 | 4-(1-pyrrolidinyl)-3-{[3-(1H-1,2,4-triazol-1-yl)propanoyl]amino}benzamide | [n]3(ncnc3)CCC(=O)Nc1c(ccc(c1)C(=O)N)N2CCCC2 | C16H20O2N6 | Enamine | Z228177696 |
| 410464285 | (2-hydroxy-3-methylphenyl)(4-(1-(2-hydroxyethyl)-1H-pyrazol-5-yl)piperidin-1-yl)methanone | [n]1(nccc1C2CCN(CC2)C(=O)c3c(c(ccc3)C)O)CCO | C18H23O3N3 | Enamine | Z1591483015 |
| 465742018 | G3iD | N1c2c(cccc2)CC(C1=O)CCC(=O)Nc3nc(nc(c3)C)c4ccccc4 | C23H22O2N4 | Enamine | Z1981613202 |
| 34299577 | 2-[4-(2-hydroxyphenyl)-1-piperazinyl]-N-(2-oxo-2,3-dihydro-1H-benzimidazol-5-yl)acetamide | N3(CCN(CC3)c4c(cccc4)O)CC(=O)Nc1cc2nc([nH]c2cc1)O | C19H21O3N5 | Enamine | Z46201483 |
| 113904437 | 4-[3-(2,4-dichlorophenoxy)-2-hydroxypropyl]-3,4-dihydro-2(1H)-quinoxalinone | Clc1c(ccc(c1)Cl)OCC(O)CN2CC(=O)Nc3c2cccc3 | C17H16O3N2Cl2 | Enamine | Z229097516 |
| 412955061 | 1-(3-((1H-1,2,4-triazol-1-yl)methyl)phenyl)-3-((1S,4R)-4-(hydroxymethyl)cyclopent-2-en-1-yl)urea | [n]3(ncnc3)Cc1cc(ccc1)NC(=O)N[C@H]2C[C@H](C=C2)CO | C16H19O2N5 | Enamine | Z1330787097 |
| 333459537 | 2-methoxy-5-[({[2-(1-piperidinyl)-3-pyridinyl]methyl}amino)methyl]phenol | N3(CCCCC3)c1ncccc1CNCc2cc(c(cc2)OC)O | C19H25O2N3 | Enamine | Z873292682 |
| 371572692 | 2-(3-hydroxyphenyl)-N-[(2-hydroxy-4-quinolinyl)methyl]acetamide | N(Cc2c3c(nc(c2)O)cccc3)C(=O)Cc1cc(ccc1)O | C18H16O3N2 | Enamine | Z1333667077 |
| 189540667 | 4-({[4-(1H-benzimidazol-2-yl)butanoyl]amino}methyl)benzamide | [nH]1c2c(nc1CCCC(=O)NCc3ccc(cc3)C(=O)N)cccc2 | C19H20O2N4 | Enamine | Z371372046 |
| 162288663 | methyl 4-{[({2-[(2-hydroxypropyl)amino]-1H-benzimidazol-1-yl}acetyl)amino]methyl}benzoate | [n]2(c3c(nc2NCC(O)C)cccc3)CC(=O)NCc1ccc(cc1)C(=O)OC | C21H24O4N4 | Enamine | Z25766652 |
| 430420324 | (3-((((7-bromobenzo[d][1,3]dioxol-5-yl)methyl)amino)methyl)phenyl)methanol | Brc1c2c(cc(c1)CNCc3cc(ccc3)CO)OCO2 | C16H16O3N1Br1 | Enamine | Z1135297588 |
| 136993869 | 2,2'-((2-chloro-4-nitrophenyl)azanediyl)bis(ethan-1-ol) | Clc1c(ccc(c1)[N+](=O)[O-])N(CCO)CCO | C10H13O4N2Cl1 | Enamine | Z1270083791 |
| 30850154 | (2E)-3-chloro-2-propenyl (2E)-3-(4-nitrophenyl)-2-propenoate | Cl\C=C\COC(=O)\C=C\c1ccc(cc1)[N+](=O)[O-] | C12H10O4N1Cl1 | Enamine | Z54849349 |
| 206911061 | N-(2-hydroxy-1-methylethyl)-N'-{[2-(4-morpholinyl)-4-pyridinyl]methyl}urea | N2(CCOCC2)c1nccc(c1)CNC(=O)NC(CO)C | C14H22O3N4 | Enamine | Z435975470 |
| 424013283 |  | [n]1(nc(nc1)c2cc(ccc2)NC(=O)NC(CO)c3ccccc3)C | C18H19O2N5 | Enamine | Z1723017494 |
| 173439436 | 1-(3-bromophenyl)-2-(4-(2-hydroxyethyl)piperazin-1-yl)ethan-1-ol | Brc1cc(ccc1)C(O)CN2CCN(CC2)CCO | C14H21O2N2Br1 | Enamine | Z320990446 |
| 371568915 | N-[3-(4-hydroxyphenyl)propyl]-4-(5-methyl-4H-1,2,4-triazol-3-yl)benzamide | [nH]1c(nnc1c2ccc(cc2)C(=O)NCCCc3ccc(cc3)O)C | C19H20O2N4 | Enamine | Z1315201787 |
| 198058947 | N-[4-(2-furoylamino)phenyl]-4-(4-hydroxyphenyl)-1-piperazinecarboxamide | N2(CCN(CC2)C(=O)Nc3ccc(cc3)NC(=O)c4[o]ccc4)c1ccc(cc1)O | C22H22O4N4 | Enamine | Z412776212 |
| 122198003 | 1-{3-fluoro-4-[4-(3-nitroimidazo[1,2-a]pyridin-2-yl)-1-piperazinyl]phenyl}ethanone | Fc1c(ccc(c1)C(=O)C)N2CCN(CC2)c3nc4[n](c3[N+](=O)[O-])C=CC=C4 | C19H18O3N5F1 | Enamine | Z128084750 |
| 947357175 |  | [S](=O)(=O)(N(C3CCc4c3cccc4)CCO)c1cc2c(cc1)CCNC2=O | C20H22O4N2S1 | Enamine | Z1497048087 |
| 39076149 | 4-{[4-(2-hydroxyphenyl)-1-piperazinyl]acetyl}-3,4-dihydro-2(1H)-quinoxalinone | N3(CCN(CC3)c4c(cccc4)O)CC(=O)N1CC(=O)Nc2c1cccc2 | C20H22O3N4 | Enamine | Z46200573 |
| 36104902 | 6-methyl-4-(3-methylphenyl)-N-(3-nitrophenyl)-2-oxo-1,2,3,4-tetrahydro-5-pyrimidinecarboxamide | [N+](=O)([O-])c1cc(ccc1)NC(=O)C2=C(NC(=O)NC2c3cc(ccc3)C)C | C19H18O4N4 | Enamine | Z47753201 |
| 122437324 | 2-methyl-4-oxo-4-[(2-oxo-2,3-dihydro-1H-benzimidazol-5-yl)amino]-2-phenylbutanoic acid | [nH]1c2c(nc1O)ccc(c2)NC(=O)CC(C)(c3ccccc3)C(=O)O | C18H17O4N3 | Enamine | Z169888110 |
| 388055917 |  | N(CCO)C(=O)c1cnc(cc1)Oc2ccccc2 | C14H14O3N2 | Enamine | Z450399074 |
| 465736254 |  | Clc1c(ccc(c1)NC(=O)[C@@H]3N(C[C@H](C3)O)C(=O)C)N2CCNC(=O)C2 | C17H21O4N4Cl1 | Enamine | Z1873287556 |
| 278495859 | 4-(6,7-dihydrothieno[3,2-c]pyridin-5(4H)-yl)-N-(4-hydroxybenzyl)-4-oxobutanamide | [s]1c2c(cc1)CN(CC2)C(=O)CCC(=O)NCc3ccc(cc3)O | C18H20O3N2S1 | Enamine | Z739437480 |
| 174641336 | N~1~-[1-(2-amino-2-oxoethyl)-4-piperidinyl]isophthalamide | N1(CCC(CC1)NC(=O)c2cc(ccc2)C(=O)N)CC(=O)N | C15H20O3N4 | Enamine | Z332853654 |
| 150770940 | (4-hydroxyphenyl)[1-(5,6,7,8-tetrahydro-4H-cyclohepta[b]thien-2-ylcarbonyl)-4-piperidinyl]methanone | [s]1c2c(cc1C(=O)N3CCC(CC3)C(=O)c4ccc(cc4)O)CCCCC2 | C22H25O3N1S1 | Enamine | Z234514874 |
| 456343845 |  | Cl.N3(CC(CC3)C(=O)O)C(=O)c1cc(ccc1)N2CCCNC2=O | C16H20O4N3Cl1 | Enamine | Z1528724129 |
| 32809290 | 4-(hydroxymethyl)-N-(2-methoxyphenyl)benzamide | N(c2c(cccc2)OC)C(=O)c1ccc(cc1)CO | C15H15O3N1 | Enamine | Z31519348 |
| 284936943 | N-(3-cyanophenyl)-N'-[2-hydroxy-3-(3-methylphenoxy)propyl]urea | N(CC(O)COc2cc(ccc2)C)C(=O)Nc1cc(ccc1)C#N | C18H19O3N3 | Enamine | Z820031172 |
| 432512029 |  | N4(CCOCC4)c1ncc(cn1)c2[nH]c3c(n2)ccc(c3)CC | C17H19O1N5 | Enamine | Z1527390066 |
| 465725654 |  | [N+](=O)([O-])c1cc(c(cc1)N)C(=O)N2CCC(CC2)(O)c3ccccc3 | C18H19O4N3 | Enamine | Z1817344290 |
| 53227621 | 2-{ethyl[(4-oxo-3,4-dihydro-2-quinazolinyl)methyl]amino}-N-[3-(1-pyrrolidinylsulfonyl)phenyl]acetamide | [S](=O)(=O)(N4CCCC4)c1cc(ccc1)NC(=O)CN(CC)C=C2Nc3c(cccc3)C(=O)N2 | C23H27O4N5S1 | Enamine | Z46301884 |
| 190039201 | methyl 3-{[3-({[(3-pyridinylmethyl)amino]carbonyl}amino)phenyl]ethynyl}benzoate | N(Cc3cnccc3)C(=O)Nc1cc(ccc1)C#Cc2cc(ccc2)C(=O)OC | C23H19O3N3 | Enamine | Z434308094 |
| 123821031 | 3-{3-[4-(4-hydroxyphenyl)-1-piperidinyl]-3-oxopropyl}-1,3-benzoxazol-2(3H)-one | N3(CCC(CC3)c4ccc(cc4)O)C(=O)CCN1c2c(cccc2)OC1=O | C21H22O4N2 | Enamine | Z237328884 |
| 947361171 |  | Fc1cc(cc(c1)CNc2c3c(ccc2)C(CCO3)O)O | C16H16O3N1F1 | Enamine | Z1715775511 |
| 45402844 | (1-{[4-amino-6-(2-ethylanilino)-1,3,5-triazin-2-yl]methyl}-1H-benzimidazol-2-yl)methanol | [n]3(c4c(nc3CO)cccc4)Cc1nc(nc(n1)N)Nc2c(cccc2)CC | C20H21O1N7 | Enamine | Z25755761 |
| 278569614 | 1-{2-[(4-nitrobenzyl)amino]phenyl}-4-piperidinol | [N+](=O)([O-])c1ccc(cc1)CNc2c(cccc2)N3CCC(CC3)O | C18H21O3N3 | Enamine | Z739800258 |
| 448919804 |  | Fc1ccc(cc1)c2[n](c(cc2)C(=O)Nc3cc(c(cc3)OC)CO)C | C20H19O3N2F1 | Enamine | Z1543192888 |
| 465720342 |  | N(C3C(c4c(cccc4)C3)O)C(=O)c1cc2c(c(c1)O)cccc2 | C20H17O3N1 | Enamine | Z1942335324 |
| 175238827 | N-[2-(1H-benzimidazol-2-yl)ethyl]-N'-[(2-methoxy-4-pyridinyl)methyl]urea | [nH]1c2c(nc1CCNC(=O)NCc3cc(ncc3)OC)cccc2 | C17H19O2N5 | Enamine | Z336588692 |
| 175950001 | N-(7,8,9,10-tetrahydro-6H-azepino[1,2-a]benzimidazol-3-yl)-1H-1,2,3-benzotriazole-5-carboxamide | [n]21c3c(nc2CCCCC1)cc(cc3)NC(=O)c4cc5nn[nH]c5cc4 | C19H18O1N6 | Enamine | Z243910642 |
| 29464398 | N4,N4'-(propane-1,3-diyl)bis(6-chloropyrimidine-2,4-diamine) | Clc1nc(nc(c1)NCCCNc2nc(nc(c2)Cl)N)N | C11H14N8Cl2 | Enamine | Z357940020 |
| 491703340 |  | [n]3(ncnc3)CC1CCN(CC1)C(=O)NCc2ccc(cc2)C(O)C | C18H25O2N5 | Enamine | Z1752308908 |
| 33350888 | N-{3-[(3,4-dihydro-2H-pyrrol-5-ylamino)sulfonyl]phenyl}-2-(1H-indol-3-yl)acetamide | [S](=O)(=O)(NC4=CCCN4)c1cc(ccc1)NC(=O)Cc2c3c([nH]c2)cccc3 | C20H20O3N4S1 | Enamine | Z27434489 |
| 295759661 | 4-{[3,5-dimethyl-4-(2-pyridinylmethoxy)benzyl]amino}cyclohexanol hydrochloride | Cl.N(C3CCC(CC3)O)Cc1cc(c(c(c1)C)OCc2ncccc2)C | C21H29O2N2Cl1 | Enamine | Z1101435909 |
| 728120205 |  | N4(CCCC4)c1c(cc(cc1)C(=O)N)NC(=O)c2cc(ncc2)OC3CCC3 | C21H24O3N4 | Enamine | Z1536578664 |
| 456174158 |  | N1c2c(cccc2)CC(C1=O)CCC(=O)NCc3cc(ccc3)C(=O)O | C20H20O4N2 | Enamine | Z1444935188 |
| 155430915 | 4-({3-[[3-([1,1'-biphenyl]-4-yloxy)-2-hydroxypropyl](ethyl)amino]propanoyl}amino)benzamide | N(CC(O)COc2ccc(cc2)c3ccccc3)(CCC(=O)Nc1ccc(cc1)C(=O)N)CC | C27H31O4N3 | Enamine | Z295847238 |
| 121552236 | 1-{2-[4-(4-nitrophenyl)-1-piperazinyl]-2-oxoethyl}-4-piperidinol | [N+](=O)([O-])c1ccc(cc1)N2CCN(CC2)C(=O)CN3CCC(CC3)O | C17H24O4N4 | Enamine | Z46457462 |
| 52971360 | (2E)-3-[1-(1,3-benzodioxol-5-yl)-2,5-dimethyl-1H-pyrrol-3-yl]-2-propenoic acid | [n]3(c(c(cc3C)\C=C\C(=O)O)C)c1cc2c(cc1)OCO2 | C16H15O4N1 | Enamine | Z2312400221 |
| 491725655 |  | N1C(CC(C1)O)C(=O)NCCOc2cc3c(cc2)NC(=O)CC3 | C16H21O4N3 | Enamine | Z2242893425 |
| 32246862 | 2-[4-(1H-indol-3-ylmethyl)-2,5-dioxo-1-imidazolidinyl]-N-(5-methyl-3-isoxazolyl)acetamide | N1C(C(=O)N(C1=O)CC(=O)Nc4n[o]c(c4)C)Cc2c3c([nH]c2)cccc3 | C18H17O4N5 | Enamine | Z16202326 |
| 386800439 | N-(2,6-dimethyl-3-nitrophenyl)-N'-[(1S,4R)-4-(hydroxymethyl)-2-cyclopenten-1-yl]urea | [N+](=O)([O-])c1c(c(c(cc1)C)NC(=O)N[C@H]2C[C@H](C=C2)CO)C | C15H19O4N3 | Enamine | Z1383385779 |
| 947346539 |  | Fc1c(ccc(c1)F)C4(CCC4)NC(=O)CCC2Cc3c(cccc3)NC2=O | C22H22O2N2F2 | Enamine | Z1542411934 |
| 369138690 | 4-(hydroxymethyl)-N-[3-methyl-4-(2,2,2-trifluoroethoxy)phenyl]-1-piperidinecarboxamide | FC(F)(F)COc1c(cc(cc1)NC(=O)N2CCC(CC2)CO)C | C16H21O3N2F3 | Enamine | Z1244046115 |
| 67340514 | 2-[3-(hydroxymethyl)-1-piperidinyl]-5-nitrobenzonitrile | [N+](=O)([O-])c1cc(c(cc1)N2CC(CCC2)CO)C#N | C13H15O3N3 | Enamine | Z31255411 |
| 58732066 | 2-(3-oxo-1-piperazinyl)-N-(2-phenoxyethyl)acetamide | N2(CCNC(=O)C2)CC(=O)NCCOc1ccccc1 | C14H19O3N3 | Enamine | Z89087324 |
| 580384511 |  | FC1(CN(CCC1C(=O)O)C(=O)OCC2c3c(cccc3)c4c2cccc4)F | C21H19O4N1F2 | Enamine | Z2471178201 |
| 188541593 | {3-[(2-nitrobenzyl)amino]phenyl}methanol | [N+](=O)([O-])c1c(cccc1)CNc2cc(ccc2)CO | C14H14O3N2 | Enamine | Z160002836 |
| 87818200 | 1-[(6-bromo-1,3-benzodioxol-5-yl)methyl]-5-nitroindoline | Brc1cc2c(cc1CN3CCc4c3ccc(c4)[N+](=O)[O-])OCO2 | C16H13O4N2Br1 | Enamine | Z185280080 |
| 282349277 | N-(2-butyl-1H-benzimidazol-5-yl)-3-(2-oxo-1,2,3,4-tetrahydro-3-quinolinyl)propanamide | [nH]1c2c(nc1CCCC)cc(cc2)NC(=O)CCC3Cc4c(cccc4)NC3=O | C23H26O2N4 | Enamine | Z826612728 |
| 277255523 | N-[5-(benzoylamino)-2-methylphenyl]-1H-1,2,3-benzotriazole-5-carboxamide | [nH]1nnc2c1ccc(c2)C(=O)Nc3c(ccc(c3)NC(=O)c4ccccc4)C | C21H17O2N5 | Enamine | Z654145204 |
| 388174352 | N'-[2-(cyclopentylsulfanyl)phenyl]-N-methyl-N-[2-(4H-1,2,4-triazol-4-yl)ethyl]urea | S(C3CCCC3)c1c(cccc1)NC(=O)N(CC[n]2cnnc2)C | C17H23O1N5S1 | Enamine | Z1409914073 |
| 424009541 |  | [S](=O)(=O)(N3CCCC3)c1c[n](c(c1)C(=O)NC2(CC(CC(C2)C)(C)C)CO)C | C20H33O4N3S1 | Enamine | Z1680942458 |
| 448923652 |  | Brc1ccc(cc1)C(=O)Nc2cc(ncc2)C(=O)N3CCC(CC3)O | C18H18O3N3Br1 | Enamine | Z1713545237 |
| 408609426 | N-(3-(3-cyclopropylureido)benzyl)-2-hydroxy-5,6,7,8-tetrahydroquinoline-3-carboxamide | N(C4CC4)C(=O)Nc1cc(ccc1)CNC(=O)c2c(nc3c(c2)CCCC3)O | C21H24O3N4 | Enamine | Z1167666509 |
| 276786968 | 1-{4-[(1,3-benzoxazol-2-ylamino)methyl]-2-fluorophenyl}-4-piperidinol | Fc1c(ccc(c1)CNc3nc4c([o]3)cccc4)N2CCC(CC2)O | C19H20O2N3F1 | Enamine | Z646292228 |
| 638632865 |  | [N+](=O)([O-])c1cc(c(cc1)NC2CCc3[n](c(nc3C2)C)CCO)C#N | C17H19O3N5 | Enamine | Z2582964010 |
| 414736700 |  | N([C@H](CO)c2ccccc2)C(=O)NC1CCC(CC1)CN(C)C | C18H29O2N3 | Enamine | Z1618940273 |
| 272168810 | 3-methyl-N-[2-methyl-1-({[3-oxo-3-(2-pyridinylamino)propyl]amino}carbonyl)propyl]benzamide | N(C(C(C)C)C(=O)NCCC(=O)Nc2ncccc2)C(=O)c1cc(ccc1)C | C21H26O3N4 | Enamine | Z649192748 |
| 209807597 | N-[3-chloro-4-(3-oxo-1-piperazinyl)phenyl]-N'-[2-(diethylamino)-2-(3-thienyl)ethyl]urea | [s]1cc(cc1)C(N(CC)CC)CNC(=O)Nc2cc(c(cc2)N3CCNC(=O)C3)Cl | C21H28O2N5Cl1S1 | Enamine | Z436056318 |
| 388183984 | 2-{[(ethylamino)carbonyl]amino}-N-[4-(1H-1,2,4-triazol-1-ylmethyl)phenyl]hexanamide | [n]2(ncnc2)Cc1ccc(cc1)NC(=O)C(NC(=O)NCC)CCCC | C18H26O2N6 | Enamine | Z1465580521 |
| 424007555 |  | Brc1c(cc(cc1)NC(=O)N(C(C)C)CCO)[N+](=O)[O-] | C12H16O4N3Br1 | Enamine | Z1671631391 |
| 330917358 | 2-[(6-fluoro-2-oxo-1,2,3,4-tetrahydro-7-quinolinyl)amino]-N-[4-(1-pyrrolidinylcarbonyl)benzyl]acetamide | Fc1cc2c(cc1NCC(=O)NCc3ccc(cc3)C(=O)N4CCCC4)NC(=O)CC2 | C23H25O3N4F1 | Enamine | Z368764614 |
| 280402933 | (4-hydroxyphenyl)(1-{[1-(2-thienylmethyl)-1H-pyrrol-2-yl]carbonyl}-4-piperidinyl)methanone | [s]1c(ccc1)C[n]2c(ccc2)C(=O)N3CCC(CC3)C(=O)c4ccc(cc4)O | C22H22O3N2S1 | Enamine | Z756736706 |
| 271093842 | 3-(2-pyrimidinylamino)-N-(tetrahydro-3-furanylmethyl)benzamide | N(CC3COCC3)C(=O)c1cc(ccc1)Nc2ncccn2 | C16H18O2N4 | Enamine | Z384358202 |
| 1060952972 | G3iC | N4(C(CCC4)CO)c1ncnc(c1)N2CCC(CC2)Oc3ncccc3C | C20H27O2N5 | Enamine | Z2179783453 |
| 397005639 | 3-((7-aminopyrido[2,3-d]pyrimidin-4-yl)amino)-1-phenylpropan-1-ol | N(CCC(O)c3ccccc3)c1ncnc2nc(ccc21)N | C16H17O1N5 | Enamine | Z1537163093 |
| 277243498 | methyl 4-{[(2-quinolinylcarbonyl)amino]methyl}phenylcarbamate | N(Cc3ccc(cc3)NC(=O)OC)C(=O)c1nc2c(cc1)cccc2 | C19H17O3N3 | Enamine | Z653909414 |
| 288624885 | N~1~-[2-(2-chlorophenyl)ethyl]-1,4-piperidinedicarboxamide | Clc1c(cccc1)CCNC(=O)N2CCC(CC2)C(=O)N | C15H20O2N3Cl1 | Enamine | Z900786504 |
| 394352523 | 1-(4-isopropylphenyl)-4-((1-(4-(pyrimidin-5-yl)phenyl)ethyl)amino)cyclohexan-1-ol | N(C3CCC(CC3)(O)c4ccc(cc4)C(C)C)C(C)c1ccc(cc1)c2cncnc2 | C27H33O1N3 | Enamine | Z1566395319 |
| 423926351 |  | [N+](=O)([O-])c1c(cc(cc1)N2CC(CCC2)Cc3[n](ccn3)C)C#N | C17H19O2N5 | Enamine | Z1696212670 |
| 331004788 | (2E)-7-(2-chlorophenyl)-2-(3-hydroxypropylidene)-N-(2-methoxyphenyl)-5-methyl-1,2,3,7-tetrahydro[1,2,4]triazolo[1,5-a]pyrimidine-6-carboxamide | Clc1c(cccc1)C2N3NC(=CCCO)NC3=NC(=C2C(=O)Nc4c(cccc4)OC)C | C23H24O3N5Cl1 | Enamine | Z1203159345 |
| 273214676 | 1-(2-methyl-3-furoyl)-4-(3-nitrophenyl)piperazine | [N+](=O)([O-])c1cc(ccc1)N2CCN(CC2)C(=O)c3c([o]cc3)C | C16H17O4N3 | Enamine | Z435259602 |
| 402233903 |  | N1(C(CN(CC1)C(=O)Cc2ccc(cc2)NC(=O)OC(C)(C)C)CC)CC(O)C | C22H35O4N3 | Enamine | Z1274936224 |
| 269375914 | 4-chloro-N-(4-hydroxybenzyl)-2-(2-oxopyrrolidin-1-yl)benzamide | Clc1cc(c(cc1)C(=O)NCc3ccc(cc3)O)N2CCCC2=O | C18H17O3N2Cl1 | Enamine | Z296528678 |
| 276786988 | 1-(4-{[(4,6-dimethyl-2-pyrimidinyl)amino]methyl}-2-fluorophenyl)-4-piperidinol | Fc1c(ccc(c1)CNc3nc(cc(n3)C)C)N2CCC(CC2)O | C18H23O1N4F1 | Enamine | Z646292356 |
| 282383188 | 1-[3-(5-phenyl-1H-pyrazol-1-yl)benzoyl]-4-piperidinecarboxamide | [n]3(nccc3c4ccccc4)c1cc(ccc1)C(=O)N2CCC(CC2)C(=O)N | C22H22O2N4 | Enamine | Z827939870 |
| 286299979 | 2-hydroxy-N-[2-hydroxy-3-(1-phenylethoxy)propyl]nicotinamide | N(CC(O)COC(C)c2ccccc2)C(=O)c1c(nccc1)O | C17H20O4N2 | Enamine | Z845218592 |
| 394757917 |  | N(CCc3ccc(cc3)O)C(=O)Nc1cc(ccc1)c2nc(c[o]2)C | C19H19O3N3 | Enamine | Z1508524368 |
| 428022475 |  | [N+](=O)([O-])c1cc(ccc1)C(Nc2cc(ccc2)N3CCCNC3=O)C | C18H20O3N4 | Enamine | Z1612880154 |
| 333516445 | N-(2-hydroxyethyl)-2-[4-(8-quinolinylmethoxy)phenyl]acetamide | N(CCO)C(=O)Cc1ccc(cc1)OCc2c3ncccc3ccc2 | C20H20O3N2 | Enamine | Z1171346571 |
| 95595555 | 4-({[4-(3,4-difluorophenyl)-4-methyl-2,5-dioxo-1-imidazolidinyl]acetyl}amino)benzamide | Fc1c(ccc(c1)C2(NC(=O)N(C2=O)CC(=O)Nc3ccc(cc3)C(=O)N)C)F | C19H16O4N4F2 | Enamine | Z134712924 |
| 506137548 |  | N3([C@@H](C[C@@H](C3)O)CO)C(=O)NCC1CN(CC1)c2c(cccc2)OC | C18H27O4N3 | Enamine | Z1734333849 |
| 947527696 |  | [n]4(ncnc4)Cc1c(cccc1)NC(=O)N[C@H]2[C@@H](C2(C)C)c3ccccc3 | C21H23O1N5 | Enamine | Z2708871873 |
| 284337275 | 1,1'-(cyclohexane-1,4-diyl)bis(3-(pyridin-4-yl)urea) | N(C2CCC(CC2)NC(=O)Nc3ccncc3)C(=O)Nc1ccncc1 | C18H22O2N6 | Enamine | Z813059112 |
| 213142131 | N-[4-(3-oxo-1-piperazinyl)phenyl]-3-(1-pyrrolidinyl)-1-piperidinecarboxamide | N4(CCCC4)C1CN(CCC1)C(=O)Nc2ccc(cc2)N3CCNC(=O)C3 | C20H29O2N5 | Enamine | Z595295632 |
| 408599956 | 4-(4-chlorophenyl)-2-(4-(4-nitrophenyl)piperazin-1-yl)quinazoline | Clc1ccc(cc1)c2nc(nc5c2cccc5)N3CCN(CC3)c4ccc(cc4)[N+](=O)[O-] | C24H20O2N5Cl1 | Enamine | Z1664285856 |
| 428057735 |  | N2(C(CC(C2)CNC(=O)c3cc(cc(c3)O)O)C)Cc1ccccc1 | C20H24O3N2 | Enamine | Z1740429081 |
| 333481095 | 4-acetyl-3-ethyl-5-methyl-N-{2-[(6-methyl-3-pyridazinyl)amino]ethyl}-1H-pyrrole-2-carboxamide | [nH]1c(c(c(c1C(=O)NCCNc2nnc(cc2)C)CC)C(=O)C)C | C17H23O2N5 | Enamine | Z1121641814 |
| 240683209 | N-[3-(aminocarbonyl)-4-chlorophenyl]-2-fluoro-5-nitrobenzamide | Fc1c(cc(cc1)[N+](=O)[O-])C(=O)Nc2cc(c(cc2)Cl)C(=O)N | C14H9O4N3Cl1F1 | Enamine | Z424992678 |
| 1069651915 |  | N2(CCc3ncncc3C2)Cc1ccc(cc1)CO | C15H17O1N3 | Enamine | Z2843516784 |
| 507485676 |  | [nH]1nc(nc1C)C2CN(CCC2)C(=O)Nc3ccc(cc3)C(=O)N | C16H20O2N6 | Enamine | Z1419911327 |
| 298899136 | N-[1-(imidazo[1,2-a]pyridin-2-ylmethyl)-1H-pyrazol-4-yl]-N'-(3-methoxyphenyl)urea | [n]21c(nc(c2)C[n]3ncc(c3)NC(=O)Nc4cc(ccc4)OC)C=CC=C1 | C19H18O2N6 | Enamine | Z994299660 |
| 284722394 | N-[3-fluoro-4-(4-hydroxy-1-piperidinyl)benzyl]-6-hydroxynicotinamide | Fc1c(ccc(c1)CNC(=O)c3cnc(cc3)O)N2CCC(CC2)O | C18H20O3N3F1 | Enamine | Z815988602 |
| 408596148 | N-(1-(1-benzylpyrrolidin-3-yl)ethyl)-3-nitrobenzamide | [N+](=O)([O-])c1cc(ccc1)C(=O)NC(C2CN(CC2)Cc3ccccc3)C | C20H23O3N3 | Enamine | Z1618949256 |
| 388001469 | 3-(5-(2,4-dichlorophenyl)oxazol-2-yl)-N-(3-hydroxypropyl)propanamide | Clc1c(ccc(c1)Cl)c2[o]c(nc2)CCC(=O)NCCCO | C15H16O3N2Cl2 | Enamine | Z352458508 |
| 465692628 |  | N1c2c(cc(cc2)C(NC(=O)c3c(cncc3)CC)C)CCC1=O | C19H21O2N3 | Enamine | Z1551792463 |
| 240667409 | 3-(2-oxo-1,2,3,4-tetrahydro-3-quinolinyl)-N-(1H-pyrazol-4-yl)propanamide | [nH]1ncc(c1)NC(=O)CCC2Cc3c(cccc3)NC2=O | C15H16O2N4 | Enamine | Z372503644 |
| 432496363 |  | N4(CCC(CC4)O)c1c(cc(cc1)NCc2cnc3[n](ncc3c2)C(C)C)C#N | C22H26O1N6 | Enamine | Z1271998224 |
| 412961845 |  | Fc1c(cc(cc1)C(=O)N2CCC(CC2)c3[n](ncc3)CCO)OC | C18H22O3N3F1 | Enamine | Z1591484614 |
| 308380336 | 2-cyclohexyl-N-[4-(3-oxo-1-piperazinyl)benzyl]acetamide | N3(CCNC(=O)C3)c1ccc(cc1)CNC(=O)CC2CCCCC2 | C19H27O2N3 | Enamine | Z397497278 |
| 320958248 | 1-(2-chloro-4-nitrophenyl)-4-(2-pyrimidinyl)-1,4-diazepane | Clc1c(ccc(c1)[N+](=O)[O-])N2CCN(CCC2)c3ncccn3 | C15H16O2N5Cl1 | Enamine | Z1127122513 |
| 410449444 |  | [N+](=O)([O-])c1ccc(cc1)CCNC(=O)N[C@H]2C[C@H](C=C2)CO | C15H19O4N3 | Enamine | Z1330702078 |
| 432498038 | G3iF | [nH]1c2c(nc1O)ccc(c2)N(Cc4nnc([o]4)C(C)(C)C)Cc3c(cccc3)C | C22H25O2N5 | Enamine | Z1299589939 |
| 465671178 |  | Sc1[n]2c(nn1)C=CC(=C2)C(=O)NCC(C(CO)(C)C)c3ccccc3 | C19H22O2N4S1 | Enamine | Z2027666882 |
| 245475409 | 4-hydroxy-N-(2-methyl-1H-indol-5-yl)-2-sulfanyl-7-quinazolinecarboxamide | Sc1nc2c(c(n1)O)ccc(c2)C(=O)Nc3cc4c([nH]c(c4)C)cc3 | C18H14O2N4S1 | Enamine | Z423600666 |
| 448210849 |  | [n]2(nc(c(c2)CNc3ccc(cc3)C(=O)N4CCC(CC4)O)C(C)(C)C)c1ccccc1 | C26H32O2N4 | Enamine | Z1908529062 |
| 513061099 |  | [nH]1nc(nc1CCNC(=O)CNC(=O)c2ccc(cc2)C(C)(C)C)N | C17H24O2N6 | Enamine | Z2233492400 |
| 276787001 | 1-{2-fluoro-4-[(7H-purin-6-ylamino)methyl]phenyl}-4-piperidinol | Fc1c(ccc(c1)CNc3ncnc4nc[nH]c43)N2CCC(CC2)O | C17H19O1N6F1 | Enamine | Z646292484 |
| 388696493 |  | Fc1c(c(cc(c1)F)c3ccccc3)NC(=O)NCCc2[nH]cnn2 | C17H15O1N5F2 | Enamine | Z1430922631 |
| 414755516 | 5-(4-(cyclobutanecarbonyl)piperazin-1-yl)-2-nitrobenzonitrile | [N+](=O)([O-])c1c(cc(cc1)N2CCN(CC2)C(=O)C3CCC3)C#N | C16H18O3N4 | Enamine | Z1696152378 |
| 448204474 |  | [n]21c(ncc2CNc3cc(ccc3)CO)NC(=C)C=C1C | C16H18O1N4 | Enamine | Z1832181984 |
| 371575179 | N-{3-[(3,5-dimethyl-1H-1,2,4-triazol-1-yl)methyl]phenyl}-N'-(4-hydroxy-1-phenylbutyl)urea | [n]3(nc(nc3C)C)Cc1cc(ccc1)NC(=O)NC(CCCO)c2ccccc2 | C22H27O2N5 | Enamine | Z1352781421 |
| 245437584 | 2-hydroxy-N-[4-(3-oxo-1-piperazinyl)phenyl]nicotinamide | N3(CCNC(=O)C3)c1ccc(cc1)NC(=O)c2c(nccc2)O | C16H16O3N4 | Enamine | Z381681324 |
| 947507947 |  | Brc1nc(cc(c1)C(=O)N[C@@H]2CC[C@@H](CC2)CO)N | C13H18O2N3Br1 | Enamine | Z2732692877 |
| 414752960 | 1-(2-(((1-(p-tolyl)-3-(1H-1,2,4-triazol-1-yl)propyl)amino)methyl)benzyl)piperidin-4-ol | [n]4(ncnc4)CCC(NCc2c(cccc2)CN3CCC(CC3)O)c1ccc(cc1)C | C25H33O1N5 | Enamine | Z1682975805 |
| 28500875 | (2E)-3-[3-nitro-4-(1-pyrrolidinyl)phenyl]-2-propenoic acid | [N+](=O)([O-])c1c(ccc(c1)\C=C\C(=O)O)N2CCCC2 | C13H14O4N2 | Key Organics / BIONET | AS-9174 |
| 27089271 | 4'-nitro[1,1'-biphenyl]-4-carboxylic acid | [N+](=O)([O-])c1ccc(cc1)c2ccc(cc2)C(=O)O | C13H9O4N1 | Key Organics / BIONET | 3X-0716 |
| 34714838 | N-(2-hydroxy-1,1-dimethylethyl)-3-{[(4-methylphenyl)sulfonyl]amino}benzamide | [S](=O)(=O)(Nc2cc(ccc2)C(=O)NC(CO)(C)C)c1ccc(cc1)C | C18H22O4N2S1 | ChemBridge Corporation | 7645593 |
| 48620803 | 3-(5-hydroxy-1H-indol-3-yl)-2-(2-(5-hydroxy-4,7-dimethyl-2-oxo-2H-chromen-3-yl)acetamido)propanoic acid | [nH]1c2c(c(c1)CC(NC(=O)CC3=C(c4c(cc(cc4O)C)OC3=O)C)C(=O)O)cc(cc2)O | C24H22O7N2 | InterBioScreen Ltd. | STOCK1N-55719 |
| 458459101 | N-(3-([1,2,4]triazolo[4,3-a]pyridin-3-yl)propyl)-4-(4-oxo-3,4-dihydroquinazolin-2-yl)butanamide | [n]21c(nnc2CCCNC(=O)CCC=C3Nc4c(cccc4)C(=O)N3)C=CC=C1 | C21H22O2N6 | InterBioScreen Ltd. | STOCK7S-53456 |
| 128071563 | 1-(3-(2-(4-oxo-3,4-dihydrophthalazin-1-yl)acetamido)propanoyl)piperidine-4-carboxamide | N3(CCC(CC3)C(=O)N)C(=O)CCNC(=O)C=C1NNC(=O)c2c1cccc2 | C19H23O4N5 | InterBioScreen Ltd. | STOCK6S-44863 |
| 458459477 | (S)-1'-ethyl-N-(1-hydroxy-3-phenylpropan-2-yl)-3',5'-dimethyl-1H,1'H-[3,4'-bipyrazole]-5-carboxamide | [n]1(nc(c(c1C)c2n[nH]c(c2)C(=O)N[C@H](CO)Cc3ccccc3)C)CC | C20H25O2N5 | InterBioScreen Ltd. | STOCK7S-54016 |
| 432301229 | 2-((S)-1-(2-(1H-indol-3-yl)ethyl)-2,5-dioxoimidazolidin-4-yl)-N-((S)-1-hydroxy-3-phenylpropan-2-yl)acetamide | N1[C@H](C(=O)N(C1=O)CCc3c4c([nH]c3)cccc4)CC(=O)N[C@H](CO)Cc2ccccc2 | C24H26O4N4 | InterBioScreen Ltd. | STOCK1N-85686 |
| 432303415 | N-((2S,3R)-1-hydroxy-3-methylpentan-2-yl)-5-oxo-1-phenethylpyrrolidine-3-carboxamide | N2(CC(CC2=O)C(=O)N[C@@H]([C@@H](CC)C)CO)CCc1ccccc1 | C19H28O3N2 | InterBioScreen Ltd. | STOCK7S-48046 |
| 363695433 | N-(3-fluoro-4-methylphenyl)-2-[(8-fluoro-5H-pyrimido[5,4-b]indol-4-yl)thio]acetamide | [S](=O)(=O)(N4CCCC4)c1cc2c(cc1)N(C(=O)CCC2)CC(=O)N3CCCCC3 | C21H29O4N3S1 | ChemDiv, Inc | L707-0212 |
| 117441101 | 3-[3-(3-fluorobenzyl)-2-oxotetrahydropyrimidin-1(2H)-yl]-N-[3-(1-hydroxyethyl)phenyl]benzamide | Fc1cc(ccc1)CN2CCCN(C2=O)c3cc(ccc3)C(=O)Nc4cc(ccc4)C(O)C | C26H26O3N3F1 | ChemDiv, Inc | J077-1129 |
| 70012770 | N~1~-cyclopropyl-N~1~-[2-(dimethylamino)-5-({[4-(trifluoromethyl)anilino]carbonyl}amino)benzyl]-4-fluorobenzamide | FC(F)(F)c1ccc(cc1)NC(=O)Nc2cc(c(cc2)N(C)C)CN(C4CC4)C(=O)c3ccc(cc3)F | C27H26O2N4F4 | ChemDiv, Inc | V025-7631 |
| 119042738 | N-(2-methylphenyl)-N'-{4-[2-(1-phenyl-1H-1,3-benzimidazol-2-yl)ethyl]phenyl}urea | [n]2(c3c(nc2CCc4ccc(cc4)NC(=O)Nc5c(cccc5)C)cccc3)c1ccccc1 | C29H26O1N4 | ChemDiv, Inc | L365-3556 |
| 234373176 | 4-[4-[(2-chlorobenzoyl)amino]-2-(morpholinocarbonyl)phenyl]-N~1~-(3-methylphenyl)tetrahydro-1(2H)-pyrazinecarboxamide | Clc1c(cccc1)C(=O)Nc2cc(c(cc2)N4CCN(CC4)C(=O)Nc5cc(ccc5)C)C(=O)N3CCOCC3 | C30H32O4N5Cl1 | ChemDiv, Inc | V005-6703 |
| 306910544 | N~3~-(cyclopropylmethyl)-N~1~-[4-({[(4-methylphenyl)amino]carbonyl}amino)phenyl]piperidine-1,3-dicarboxamide | N3(CC(CCC3)C(=O)NCC4CC4)C(=O)Nc1ccc(cc1)NC(=O)Nc2ccc(cc2)C | C25H31O3N5 | ChemDiv, Inc | M667-1035 |
| 117515708 | N-(2-chlorophenyl)-3-{5-methyl-2-[(4-methylbenzyl)amino]-7-oxo-4,7-dihydro[1,2,4]triazolo[1,5-a]pyrimidin-6-yl}propanamide | Clc1c(cccc1)NC(=O)CCC2=C(Nc3[n](nc(n3)NCc4ccc(cc4)C)C2=O)C | C23H23O2N6Cl1 | ChemDiv, Inc | D481-0126 |
| 111440870 | N-{3-[benzyl(methyl)amino]propyl}-5-[(diethylamino)sulfonyl]-1-methyl-1H-indole-2-carboxamide | [S](=O)(=O)(N(CC)CC)c1cc2c([n](c(c2)C(=O)NCCCN(Cc3ccccc3)C)C)cc1 | C25H34O3N4S1 | ChemDiv, Inc | G732-0231 |
| 117514269 | N-(4-{[(4-{[6-(dimethylamino)-2-methylpyrimidin-4-yl]amino}phenyl)amino]sulfonyl}phenyl)acetamide (G3iA) | [S](=O)(=O)(Nc2ccc(cc2)Nc3nc(nc(c3)N(C)C)C)c1ccc(cc1)NC(=O)C | C21H24O3N6S1 | ChemDiv, Inc | D430-1191 |
| 234332233 | N-(2,3-dichlorophenyl)-N'-{3-[(4-methylpiperidino)carbonyl]-4-piperidinophenyl}urea | Clc1c(cccc1NC(=O)Nc2cc(c(cc2)N4CCCCC4)C(=O)N3CCC(CC3)C)Cl | C25H30O2N4Cl2 | ChemDiv, Inc | V014-2647 |
| 90324129 | 1-[(6-chloro-1,3-benzodioxol-5-yl)methyl]-4-(3-chloro-4-fluorophenyl)-1H-indol-5-yl N,N-diethylcarbamate | Fc1c(cc(cc1)c2c3c([n](cc3)Cc4cc5c(cc4Cl)OCO5)ccc2OC(=O)N(CC)CC)Cl | C27H23O4N2Cl2F1 | ChemDiv, Inc | V008-9620 |
| 117418266 | N-(3-methylphenyl)-N'-{2-[5-(piperidin-1-ylcarbonyl)-1H-1,2,3-benzotriazol-1-yl]ethyl}urea | N4(CCCCC4)C(=O)c1cc2nn[n](c2cc1)CCNC(=O)Nc3cc(ccc3)C | C22H26O2N6 | ChemDiv, Inc | G373-3493 |
| 339171808 | 2-(4-oxo-3,4-dihydro-1-phthalazinyl)-N-[4-(1H-1,2,4-triazol-1-ylmethyl)phenyl]acetamide | [n]4(ncnc4)Cc1ccc(cc1)NC(=O)C=C2NNC(=O)c3c2cccc3 | C19H16O2N6 | ChemDiv, Inc | Y043-3098 |
| 120984885 | N-(2-hydroxypropyl)-N'-{4-[4-({[(2-hydroxypropyl)(methyl)amino]carbonyl}amino)benzyl]phenyl}-N-methylurea | N(CC(O)C)(C)C(=O)Nc1ccc(cc1)Cc2ccc(cc2)NC(=O)N(CC(O)C)C | C23H32O4N4 | Labotest | LT03506200 |
| 947226997 |  | Clc1ccc(cc1)C2(CCN(CC2)C(=O)CCCC(=O)N3CCNC(=O)C3)O | C20H26O4N3Cl1 | Pharmeks LTD. | PHAR367090 |
| 61813241 | N-(2,2,6,6-tetramethyl-4-piperidinyl)-2-(3,4,9-trimethyl-7-oxo-7H-furo[2,3-f]chromen-8-yl)acetamide | N1C(CC(CC1(C)C)NC(=O)CC2=C(c3c4[o]cc(c4c(cc3OC2=O)C)C)C)(C)C | C25H32O4N2 | Pharmeks LTD. | PHAR200874 |
| 457515381 |  | [n]1(c2c(cc1)cc(cc2)NC(=O)CCCc3nc4c(c(n3)O)cccc4)C | C21H20O2N4 | Vitas-M Laboratory, Ltd. | STL422662 |
| 128086241 | N-(2-hydroxyethyl)-Nalpha-{[4-(1H-indol-2-ylcarbonyl)piperazin-1-yl]carbonyl}-L-phenylalaninamide | N2(CCN(CC2)C(=O)c3[nH]c4c(c3)cccc4)C(=O)N[C@@H](Cc1ccccc1)C(=O)NCCO | C25H29O4N5 | Vitas-M Laboratory, Ltd. | STK618830 |
| 113782447 | trans-N-[(2S)-1-{[2-(dimethylamino)ethyl]amino}-1-oxopropan-2-yl]-4-{[(phenylsulfonyl)amino]methyl}cyclohexanecarboxamide | [S](=O)(=O)(NC[C@@H]2CC[C@H](CC2)C(=O)N[C@@H](C)C(=O)NCCN(C)C)c1ccccc1 | C21H34O4N4S1 | Vitas-M Laboratory, Ltd. | STK626627 |
| 28875055 | 4,4'-oxybis(N-(3-iodophenyl)benzamide) | Ic1cc(ccc1)NC(=O)c2ccc(cc2)Oc3ccc(cc3)C(=O)Nc4cc(ccc4)I | C26H18O3N2I2 | Vitas-M Laboratory, Ltd. | STK058635 |
| 28412375 | 4-(2-chloro-5-nitrophenyl)-6,7-dimethyl-3,4-dihydroquinolin-2(1H)-one | Clc1c(cc(cc1)[N+](=O)[O-])C2CC(=O)Nc3c2cc(c(c3)C)C | C17H15O3N2Cl1 | Vitas-M Laboratory, Ltd. | STK003156 |
| 53080361 | N-(2,4-dimethylphenyl)-6-({4-[(2E)-3-phenylprop-2-en-1-yl]piperazin-1-yl}methyl)-1,3,5-triazine-2,4-diamine | N3(CCN(CC3)C\C=C\c4ccccc4)Cc1nc(nc(n1)N)Nc2c(cc(cc2)C)C | C25H31N7 | Vitas-M Laboratory, Ltd. | STK203554 |
| 90161350 | 1-(4-methoxybenzyl)-3-(4-methyl-6-oxo-1,6-dihydropyrimidin-2-yl)guanidine | N(Cc2ccc(cc2)OC)C(=N)Nc1nc(cc(n1)C)O | C14H17O2N5 | Vitas-M Laboratory, Ltd. | STK263579 |
| 27577368 | 4-(2-chloro-4-nitrophenyl)-2-methyl-1-(phenylsulfonyl)piperazine | [S](=O)(=O)(N2C(CN(CC2)c3c(cc(cc3)[N+](=O)[O-])Cl)C)c1ccccc1 | C17H18O4N3Cl1S1 | Vitas-M Laboratory, Ltd. | STK148711 |
| 34652698 | N-(4-(benzyloxy)phenyl)-2-(2-(4-chlorophenylamino)-2-oxoethoxy)acetamide | Clc1ccc(cc1)NC(=O)COCC(=O)Nc2ccc(cc2)OCc3ccccc3 | C23H21O4N2Cl1 | Vitas-M Laboratory, Ltd. | STK250299 |
| 458459064 |  | [n]1(c2c(nc1C)cc(cc2)N3C=C4C(=NNC4=O)C(=C3)C(=O)OC)CCO | C18H17O4N5 | Vitas-M Laboratory, Ltd. | STL493652 |
| 258703945 | 3-({2-[4-(2-hydroxyethyl)-1-piperazinyl]-4-quinazolinyl}amino)-1-propanol | N1(CCN(CC1)c2nc3c(c(n2)NCCCO)cccc3)CCO | C17H25O2N5 | Vitas-M Laboratory, Ltd. | STL156365 |
| 29353698 | 3-{[6-bromo-4-(2-chlorophenyl)quinazolin-2-yl]amino}propan-1-ol | Brc1cc2c(nc(nc2c3c(cccc3)Cl)NCCCO)cc1 | C17H15O1N3Cl1Br1 | Vitas-M Laboratory, Ltd. | STK842849 |
| 65080771 | 3-(5-chloro-2-hydroxy-4-methylphenyl)-5-(2-hydroxyethyl)-4-[4-(propan-2-yl)phenyl]-4,5-dihydropyrrolo[3,4-c]pyrazol-6(2H)-one | Clc1c(cc(c(c1)c2[nH]nc3c2C(N(C3=O)CCO)c4ccc(cc4)C(C)C)O)C | C23H24O3N3Cl1 | Vitas-M Laboratory, Ltd. | STK588049 |
| 38991555 | 6-bromo-2-[(E)-2-(4-hydroxyphenyl)ethenyl]-3-(2-methoxyphenyl)quinazolin-4(3H)-one (G3iB) | Brc1cc2c(cc1)N=C(N(C2=O)c4c(cccc4)OC)\C=C\c3ccc(cc3)O | C23H17O3N2Br1 | Vitas-M Laboratory, Ltd. | STL250579 |
| 119562983 | 1-(7-chloro-2,3-dimethyl-1H-indol-1-yl)-3-[4-(2-hydroxyethyl)-1-piperazinyl]-2-propanol | Clc1c2[n](c(c(c2ccc1)C)C)CC(O)CN3CCN(CC3)CCO | C19H28O2N3Cl1 | Vitas-M Laboratory, Ltd. | STK944943 |
| 29553391 | 4-[(diphenylacetyl)oxy]benzoic acid | O(c3ccc(cc3)C(=O)O)C(=O)C(c2ccccc2)c1ccccc1 | C21H16O4 | Vitas-M Laboratory, Ltd. | STK545575 |
| 26879615 | 3-(4-nitrobenzyl)-1,3-benzoxazol-2(3H)-one | [N+](=O)([O-])c1ccc(cc1)CN2c3c(cccc3)OC2=O | C14H10O4N2 | Vitas-M Laboratory, Ltd. | STK735423 |
| 37495013 | 2-[4-(8-nitroquinolin-4-yl)piperazin-1-yl]ethanol | [N+](=O)([O-])c1c2nccc(c2ccc1)N3CCN(CC3)CCO | C15H18O3N4 | Vitas-M Laboratory, Ltd. | STK198623 |
| 29350280 | 6-methyl-2-[4-(4-nitrophenyl)piperazin-1-yl]-4-phenylquinazoline | [N+](=O)([O-])c1ccc(cc1)N2CCN(CC2)c3nc4c(c(n3)c5ccccc5)cc(cc4)C | C25H23O2N5 | Vitas-M Laboratory, Ltd. | STK841545 |
| 31275424 | methyl (2E)-3-(4-nitrophenyl)-2-phenylprop-2-enoate | [N+](=O)([O-])c1ccc(cc1)\C=C(/c2ccccc2)\C(=O)OC | C16H13O4N1 | Vitas-M Laboratory, Ltd. | STK663970 |
| 27221431 | 2,2'-(9-methyl-9H-carbazole-3,6-diyl)diacetic acid | [n]1(c2c(c3c1ccc(c3)CC(=O)O)cc(cc2)CC(=O)O)C | C17H15O4N1 | Vitas-M Laboratory, Ltd. | STK530424 |
| 30005010 | N-benzyl-1-(3-ethoxy-4-hydroxybenzyl)-N-(2-hydroxyethyl)-4-piperidinecarboxamide | N2(CCC(CC2)C(=O)N(CCO)Cc3ccccc3)Cc1cc(c(cc1)O)OCC | C24H32O4N2 | Vitas-M Laboratory, Ltd. | STK025085 |
| 61578219 | N-{2-[1-(3-chlorobenzyl)-1H-benzimidazol-2-yl]ethyl}-4-hydroxypentanamide | Clc1cc(ccc1)C[n]2c3c(nc2CCNC(=O)CCC(O)C)cccc3 | C21H24O2N3Cl1 | Vitas-M Laboratory, Ltd. | STK739223 |
| 374589698 | N-(2-hydroxyethyl)-4-(2-pyrimidinylamino)benzamide | N(CCO)C(=O)c1ccc(cc1)Nc2ncccn2 | C13H14O2N4 | Vitas-M Laboratory, Ltd. | STL317561 |
| 28736975 | 1-(1,4-dioxaspiro[4.5]dec-2-ylmethyl)-1-[4-(methoxycarbonyl)benzyl]pyrrolidinium | [N+]4(CCCC4)(CC2OC3(OC2)CCCCC3)Cc1ccc(cc1)C(=O)OC | C22H32O4N1 | Vitas-M Laboratory, Ltd. | STK010398 |
| 62284998 | 1-({4-amino-6-[(4-ethylphenyl)amino]-1,3,5-triazin-2-yl}methyl)piperidine-4-carboxamide | N3(CCC(CC3)C(=O)N)Cc1nc(nc(n1)N)Nc2ccc(cc2)CC | C18H25O1N7 | Vitas-M Laboratory, Ltd. | STK231489 |
| 113775287 | 2-{(1E)-5-fluoro-2-methyl-1-[4-(methylsulfinyl)benzylidene]-1H-inden-3-yl}-N-(3-hydroxyphenyl)acetamide | Fc1cc2c(cc1)\C(=C\c4ccc(cc4)[S](=O)C)\C(=C2CC(=O)Nc3cc(ccc3)O)C | C26H22O3N1S1F1 | Vitas-M Laboratory, Ltd. | STL008997 |
| 38209864 | 1-(4-methoxy-2-nitrophenyl)-4-(2-methylbenzoyl)piperazine | [N+](=O)([O-])c1c(ccc(c1)OC)N2CCN(CC2)C(=O)c3c(cccc3)C | C19H21O4N3 | Vitas-M Laboratory, Ltd. | STK206470 |
| 37459199 | N-[1-(2,6-dichlorobenzyl)-1H-pyrazol-3-yl]-N'-[2-(4-nitro-1H-pyrazol-1-yl)ethyl]thiourea | S=C(NCC[n]3ncc(c3)[N+](=O)[O-])Nc1n[n](cc1)Cc2c(cccc2Cl)Cl | C16H15O2N7Cl2S1 | Vitas-M Laboratory, Ltd. | STK311594 |
| 68523562 | N-{3-[(2-chlorobenzyl)amino]-3-oxopropyl}-3-hydroxyquinoxaline-1(2H)-carboxamide | Clc1c(cccc1)CNC(=O)CCNC(=O)N2CC(=O)Nc3c2cccc3 | C19H19O3N4Cl1 | Vitas-M Laboratory, Ltd. | STK613090 |
| 29648851 | N-{3-[(3-chloroanilino)carbonyl]phenyl}-3-nitrobenzamide (G3iE) | Clc1cc(ccc1)NC(=O)c2cc(ccc2)NC(=O)c3cc(ccc3)[N+](=O)[O-] | C20H14O4N3Cl1 | Vitas-M Laboratory, Ltd. | STL069587 |
| 40573444 | {4-[4-nitro-2-(trifluoromethyl)phenyl]piperazin-1-yl}(thiophen-2-yl)methanone | FC(F)(F)c1c(ccc(c1)[N+](=O)[O-])N2CCN(CC2)C(=O)c3[s]ccc3 | C16H14O3N3S1F3 | Vitas-M Laboratory, Ltd. | STK205979 |
| 30169635 | 2-(4-{3-[4-(2-methylquinolin-4-yl)piperazin-1-yl]-4-nitrophenyl}piperazin-1-yl)ethanol | [N+](=O)([O-])c1c(cc(cc1)N5CCN(CC5)CCO)N2CCN(CC2)c3c4c(nc(c3)C)cccc4 | C26H32O3N6 | Vitas-M Laboratory, Ltd. | STK150205 |
| 40921726 | (2,5-dichlorophenyl)[4-(4-methoxy-2-nitrophenyl)piperazin-1-yl]methanone | Clc1c(cc(cc1)Cl)C(=O)N2CCN(CC2)c3c(cc(cc3)OC)[N+](=O)[O-] | C18H17O4N3Cl2 | Vitas-M Laboratory, Ltd. | STK172629 |

**Supplementary Table S2. IC_50_ values for GLUT3 inhibitors in wild-type and S66Y GLUT3**. Dose response curves are in Figure 2 for wild-type GLUT3 and Supplementary Figure S3 for GLUT3_S66Y_.

|  | IC_50_ (µM) | | | | | |
| --- | --- | --- | --- | --- | --- | --- |
|  | G3iA | G3iB | G3iC | G3iD | G3iE | G3iF |
| GLUT3 | 7.01 ± 1.02 | 12.2 ± 4.0 | 12.5 ±4.4 | 19.3 ± 4.7 | 23.6 ± 1.9 | 38.4 ± 11.8 |
| GLUT3_S66Y_ | 6.21 ± 0.95 | 9.62 ± 1.17 | 10.1 ± 2.1 | 12.6 ± 1.6 | 20.6 ± 2.4 | 24.8 ± 6.0 |

**REFERENCES**

1. Schmidl, S., Tamayo Rojas, S. A., Iancu, C. V., Choe, J.-Y. & Oreb, M. Functional Expression of the Human Glucose Transporters GLUT2 and GLUT3 in Yeast Offers Novel Screening Systems for GLUT-Targeting Drugs. *Front Mol Biosci* **7**, 598419 (2021).

2. Dixon, M. The determination of enzyme inhibitor constants. *Biochem J* **55**, 170–171 (1953).

3. Wieczorke, R., Dlugai, S., Krampe, S. & Boles, E. Characterisation of mammalian GLUT glucose transporters in a heterologous yeast expression system. *Cell. Physiol. Biochem.* **13**, 123–134 (2003).
